# The Bayes Estimators of the Variance and Scale Parameters of the Normal Model With a Known Mean for the Conjugate and Noninformative Priors Under Stein’s Loss

**DOI:** 10.3389/fdata.2021.763925

**Published:** 2022-01-03

**Authors:** Ying-Ying Zhang, Teng-Zhong Rong, Man-Man Li

**Affiliations:** ^1^ Department of Statistics and Actuarial Science, College of Mathematics and Statistics, Chongqing University, Chongqing, China; ^2^ Chongqing Key Laboratory of Analytic Mathematics and Applications, Chongqing University, Chongqing, China

**Keywords:** Bayes estimator, variance and scale parameters, normal model, conjugate and noninformative priors, Stein’s loss

## Abstract

For the normal model with a known mean, the Bayes estimation of the variance parameter under the conjugate prior is studied in Lehmann and Casella (1998) and Mao and Tang (2012). However, they only calculate the Bayes estimator with respect to a conjugate prior under the squared error loss function. Zhang (2017) calculates the Bayes estimator of the variance parameter of the normal model with a known mean with respect to the conjugate prior under Stein’s loss function which penalizes gross overestimation and gross underestimation equally, and the corresponding Posterior Expected Stein’s Loss (PESL). Motivated by their works, we have calculated the Bayes estimators of the variance parameter with respect to the noninformative (Jeffreys’s, reference, and matching) priors under Stein’s loss function, and the corresponding PESLs. Moreover, we have calculated the Bayes estimators of the scale parameter with respect to the conjugate and noninformative priors under Stein’s loss function, and the corresponding PESLs. The quantities (prior, posterior, three posterior expectations, two Bayes estimators, and two PESLs) and expressions of the variance and scale parameters of the model for the conjugate and noninformative priors are summarized in two tables. After that, the numerical simulations are carried out to exemplify the theoretical findings. Finally, we calculate the Bayes estimators and the PESLs of the variance and scale parameters of the S&P 500 monthly simple returns for the conjugate and noninformative priors.

## 1 Introduction

There are four basic elements in Bayesian decision theory and specifically in Bayesian point estimation: The data, the model, the prior, and the loss function. In this paper, we are interested in the data from the normal model with a known mean, with respect to the conjugate and noninformative (Jeffreys’s, reference, and matching) priors, under Stein’s and the squared error loss functions. We will analytically calculate the Bayes estimators of the variance and scale parameters of the normal model with a known mean, with respect to the conjugate and noninformative priors under Stein’s and the squared error loss functions.

The squared error loss function has been used by many authors for the problem of estimating the variance, *σ* ^2^, based on a random sample from a normal distribution (see for instance (Maatta and Casella, 1990)). As pointed out by (Casella and Berger, 2002), the squared error loss function penalizes overestimation and underestimation equally, which is fine for the location parameter with parameter space $\Theta =\left(-\infty ,\infty \right)$ . For a variance or scale parameter, the parameter space is $\Theta =\left(0,\infty \right)$ where 0 is a natural lower bound and the estimation problem is not symmetric. In these cases, we should not choose the squared error loss function, but choose a loss function which penalizes gross overestimation and gross underestimation equally, that is, an action *a* will incur an infinite loss when it tends to 0 or *∞*. Stein’s loss function has this property, and thus it is recommended to use for the positive restricted parameter space $\Theta =\left(0,\infty \right)$ by many authors (see for example (James and Stein, 1961; Petropoulos and Kourouklis, 2005; Oono and Shinozaki, 2006; Bobotas and Kourouklis, 2010; Zhang, 2017; Xie et al., 2018; Zhang et al., 2019; Sun et al., 2021)). In the normal model with a known mean *μ*, our parameters of interest are *θ* = *σ* ^2^ (a variance parameter) and *θ* = *σ* (a scale parameter). Therefore, we will select Stein’s loss function.

The motivation and contributions of our paper are summarized as follows. For the normal model with a known mean *μ*, the Bayes estimation of the variance parameter *θ* = *σ* ^2^ under the conjugate prior which is an Inverse Gamma distribution is studied in Example 4.2.5 (p.236) of (Lehmann and Casella, 1998) and Example 1.3.5 (p.15) of (Mao and Tang, 2012). However, they only calculate the Bayes estimator with respect to a conjugate prior under the squared error loss. (Zhang, 2017) calculates the Bayes estimator of the variance parameter *θ* = *σ* ^2^ of the normal model with a known mean with respect to the conjugate prior under Stein’s loss function which penalizes gross overestimation and gross underestimation equally, and the corresponding Posterior Expected Stein’s Loss (PESL). Motivated by the works of (Lehmann and Casella, 1998; Mao and Tang, 2012; Zhang, 2017), we want to calculate the Bayes estimators of the variance and scale parameters of the normal model with a known mean for the conjugate and noninformative priors under Stein’s loss function. The contributions of our paper are summarized as follows. In this paper, we have calculated the Bayes estimators of the variance parameter *θ* = *σ* ^2^ with respect to the noninformative (Jeffreys’s, reference, and matching) priors under Stein’s loss function, and the corresponding Posterior Expected Stein’s Losses (PESLs). Moreover, we have calculated the Bayes estimators of the scale parameter *θ* = *σ* with respect to the conjugate and noninformative priors under Stein’s loss function, and the corresponding PESLs. For more literature on Bayesian estimation and inference, we refer readers to (Sindhu and Aslam, 2013a; Sindhu and Aslam, 2013b; Sindhu et al., 2013; Sindhu et al., 2016a; Sindhu et al., 2016b; Sindhu et al., 2016c; Sindhu et al., 2017; Sindhu et al., 2018; Sindhu and Hussain, 2018)

The rest of the paper is organized as follows. In the next Section 2, we analytically calculate the Bayes estimators of the variance and scale parameters of the normal model with a known mean, with respect to the conjugate and noninformative priors under Stein’s loss function, and the corresponding PESLs. We also analytically calculate the Bayes estimators under the squared error loss function, and the corresponding PESLs. The quantities (prior, posterior, three posterior expectations, two Bayes estimators, and two PESLs) and expressions of the variance and scale parameters for the conjugate and noninformative priors are summarized in two tables. Section 3 reports vast amount of numerical simulation results of the combination of the noninformative prior and the scale parameter to support the theoretical studies of two inequalities of the Bayes estimators and the PESLs, and that the PESLs depend only on the number of observations, but do not depend on the mean and the sample. In Section 4, we calculate the Bayes estimators and the PESLs of the variance and scale parameters of the S&P 500 monthly simple returns for the conjugate and noninformative priors. Some conclusions and discussions are provided in Section 5.

## 2 Bayes Estimator, PESL, IRSL, and BRSL

In this section, we will analytically calculate the Bayes estimator $\delta_{s}^{\pi ,\theta }\left(x\right)$ of the variance parameter $\theta =\sigma^{2}\in \Theta =\left(0,\infty \right)$ under Stein’s loss function, the PESL at $\delta_{s}^{\pi ,\theta }\left(x\right)$ , $PESL_{s}^{\pi ,\theta }\left(x\right)$ , and the Integrated Risk under Stein’s Loss (IRSL) at $\delta_{s}^{\pi ,\theta }$ , $IRSL_{s}^{\pi ,\theta }=BRSL^{\pi ,\theta }$ , which is also the Bayes Risk under Stein’s Loss (BRSL) for *π*, *θ*. See (Robert, 2007) for the definitions of the posterior expected loss, the integrated risk, and the Bayes risk. We will also analytically calculate the Bayes estimator $\delta_{s}^{\pi ,\sigma }\left(x\right)$ of the scale parameter $\sigma \in \Theta =\left(0,\infty \right)$ under Stein’s loss function, the PESL at $\delta_{s}^{\pi ,\sigma }\left(x\right)$ , $PESL_{s}^{\pi ,\sigma }\left(x\right)$ , and the IRSL at $\delta_{s}^{\pi ,\sigma }$ , $IRSL_{s}^{\pi ,\sigma }=BRSL^{\pi ,\sigma }$ , which is also the BRSL for *π*, *σ*.

Suppose that we observe *X*_1_, *X*_2_, …, *X*_*n*_ from the hierarchical normal model with a mixing variance parameter *θ* = *σ* ^2^:

$$\left\{\begin{array}{c} X_{i}|\theta \overset{\text{iid}}{∼}N\left(\mu ,\theta \right),\;i=1,2,\ldots ,n,\;\\ \theta ∼\pi \left(\theta \right),\; \end{array}\right.$$ (1)

where − *∞* < *μ* < *∞* is a known constant, $N\left(\mu ,\theta \right)$ is the normal distribution with a known mean *μ* and an unknown variance *θ*, and $\pi \left(\theta \right)$ is the prior distribution of *θ*. For the normal model with a known mean *μ*, the Bayes estimation of the variance parameter *θ* = *σ* ^2^ under the conjugate prior which is an Inverse Gamma distribution is studied in Example 4.2.5 (p.236) of (Lehmann and Casella, 1998) and Example 1.3.5 (p.15) of (Mao and Tang, 2012). However, they only calculate the Bayes estimator with respect to a conjugate prior under the squared error loss. (Zhang, 2017) calculates the Bayes estimator of the variance parameter *θ* = *σ* ^2^ with respect to the conjugate prior under Stein’s loss function, and the corresponding PESL. Motivated by the works of (Lehmann and Casella, 1998; Mao and Tang, 2012; Zhang, 2017), we want to calculate the Bayes estimators of the variance parameter of the normal model with a known mean for the noninformative (Jeffreys’s, reference, and matching) priors under Stein’s loss function. The usual Bayes estimator with respect to a prior $\pi \left(\theta \right)$ is to calculate $\delta_{2}^{\pi ,\theta }\left(x\right)=E\left(\theta |x\right)$ under the squared error loss function. As pointed out in the introduction, we should calculate and use the Bayes estimator of the variance parameter *θ* with respect to a prior $\pi \left(\theta \right)$ under Stein’s loss function, that is, $\delta_{s}^{\pi ,\theta }\left(x\right)$ .

Alternatively, we may be interested in the scale parameter *θ* = *σ*. Motivated by the works of (Lehmann and Casella, 1998; Mao and Tang, 2012; Zhang, 2017), we also want to calculate the Bayes estimators of the scale parameter *θ* = *σ* with respect to the conjugate and noninformative priors under Stein’s loss function, and the corresponding PESLs. Suppose that we observe *X*_1_, *X*_2_, …, *X*_*n*_ from the hierarchical normal model with a mixing scale parameter *θ* = *σ*:

$$\left\{\begin{array}{c} X_{i}|\sigma \overset{\text{iid}}{∼}N\left(\mu ,\sigma^{2}\right),\;i=1,2,\ldots ,n,\;\\ \sigma ∼\pi \left(\sigma \right),\; \end{array}\right.$$ (2)

where − *∞* < *μ* < *∞* is a known constant, $N\left(\mu ,\sigma^{2}\right)$ is the normal distribution with a known mean *μ* and an unknown variance *σ* ^2^, and $\pi \left(\sigma \right)$ is the prior distribution of *σ*. The usual Bayes estimator with respect to a prior $\pi \left(\sigma \right)$ is to calculate $\delta_{2}^{\pi ,\sigma }\left(x\right)=E\left(\sigma |x\right)$ under the squared error loss function. As pointed out in the introduction, we should calculate and use the Bayes estimator of the scale parameter *σ* with respect to a prior $\pi \left(\sigma \right)$ under Stein’s loss function, that is, $\delta_{s}^{\pi ,\sigma }\left(x\right)$ .

Now let us explain why we choose Stein’s loss function on $\Theta =\left(0,\infty \right)$ . Stein’s loss function is given by

$$L_{s}\left(\theta ,a\right)=\frac{a}{\theta }-\log\frac{a}{\theta }-1,$$ (3)

where *θ* > 0 is the unknown parameter of interest and *a* is an action or estimator. The squared error loss function is given by

$$L_{2}\left(\theta ,a\right)={\left(a-\theta \right)}^{2}.$$ (4)

**FIGURE 1:**
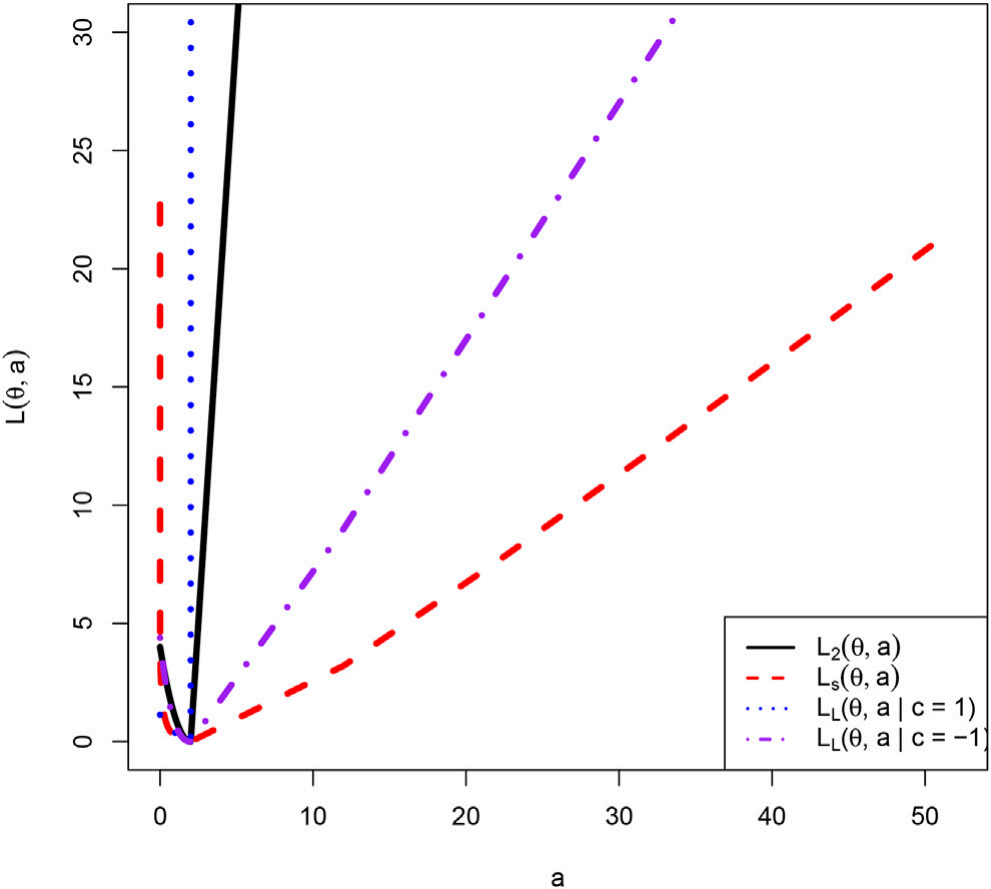
The four loss functions on $\Theta =\left(0,\infty \right)$ when *θ* = 2.

The asymmetric Linear Exponential (LINEX) loss function ((Varian et al., 1975; Zellner, 1986; Robert, 2007)) is given by

$$L_{L}\left(\theta ,a\right)=e^{c\left(a-\theta \right)}-c\left(a-\theta \right)-1,$$ (5)

where *c* ≠ 0 serving to determine its shape. In particular, when *c* > 0, the LINEX loss function tends to *∞* exponentially, while when *c* < 0, the LINEX loss function tends to *∞* linearly. Note that on the positive restricted parameter space $\Theta =\left(0,\infty \right)$ , Stein’s loss function penalizes gross overestimation and gross underestimation equally, that is, an action *a* will incur an infinite loss when it tends to 0 or *∞*. Whereas, the squared error loss function does not penalize gross overestimation and gross underestimation equally, as an action *a* will incur a finite loss (in fact *θ* ^2^) when it tends to 0 and incur an infinite loss when it tends to *∞*. Similarly, the LINEX loss functions also do not penalize gross overestimation and gross underestimation equally, as an action *a* will incur a finite loss (in fact *e* ^−*cθ*^ + *cθ* − 1) when it tends to 0 and incur an infinite loss when it tends to *∞*. Figure 1 shows the four loss functions on $\Theta =\left(0,\infty \right)$ when *θ* = 2.

As pointed out by (Zhang, 2017), the Bayes estimator

$$\delta_{s}^{\pi ,\theta }\left(x\right)=\frac{1}{E\left(\frac{1}{\theta }|x\right)}$$

minimizes the PESL, that is,

$$\delta_{s}^{\pi ,\theta }\left(x\right)=\arg\underset{a\in A}{\min}E\left[L_{s}\left(\theta ,a\right)|x\right],$$

where $\mathrm{A=}\left\{a\left(x\right):a\left(x\right)>0\right\}$ is an action space, $a=a\left(x\right)>0$ is an action (estimator), which is a function only of *x*, $L_{s}\left(\theta ,a\right)$ given by (Eq. 3) is Stein’s loss function, and *θ* > 0 is the unknown parameter of interest. Note that Stein’s loss function has a nice property that it penalizes gross overestimation and gross underestimation equally, that is, an action *a* will incur an infinite loss when it tends to 0 or *∞*. Moreover, note that *θ* may be the variance parameter *σ* ^2^ or the scale parameter *σ*.

The usual Bayes estimator of *θ* is $\delta_{2}^{\pi ,\theta }\left(x\right)=E\left(\theta |x\right)$ which minimizes the Posterior Expected Squared Error Loss. It is interesting to note that

$$\delta_{s}^{\pi ,\theta }\left(x\right)\leq \delta_{2}^{\pi ,\theta }\left(x\right),$$ (6)

whose proof exploits Jensen’s inequality and the proof can be found in (Zhang, 2017). Note that the inequality (Eq. 6) is a special inequality in (Zhang et al., 2018). As calculated in (Zhang, 2017), the PESL at $\delta_{s}^{\pi ,\theta }\left(x\right)={\left[E\left(\theta^{-1}|x\right)\right]}^{-1}$ is

$$PESL_{s}^{\pi ,\theta }\left(x\right)={\left.E\left[L_{s}\left(\theta ,a\right)|x\right]\right|}_{a=\frac{1}{E\left(\frac{1}{\theta }|x\right)}}=\log E\left(\frac{1}{\theta }|x\right)+E\left(\log\theta |x\right),$$

and the PESL at $\delta_{2}^{\pi ,\theta }\left(x\right)=E\left(\theta |x\right)$ is

$$\begin{array}{ll} PESL_{2}^{\pi ,\theta }\left(x\right) & ={\left.E\left[L_{s}\left(\theta ,a\right)|x\right]\right|}_{a=E\left(\theta |x\right)}\\ & =E\left(\theta |x\right)E\left(\frac{1}{\theta }|x\right)-\log E\left(\theta |x\right)+E\left(\log\theta |x\right)-1. \end{array}$$

As observed in (Zhang, 2017),

$$PESL_{s}^{\pi ,\theta }\left(x\right)\leq PESL_{2}^{\pi ,\theta }\left(x\right),$$ (7)

which is a direct consequence of the general methodology for finding a Bayes estimator or due to $\delta_{s}^{\pi ,\theta }\left(x\right)$ minimizes the PESL. The numerical simulations will exemplify (Eqs 6, 7) later. Note that the calculations of $\delta_{s}^{\pi ,\theta }\left(x\right)$ , $\delta_{2}^{\pi ,\theta }\left(x\right)$ , $PESL_{s}^{\pi ,\theta }\left(x\right)$ , and $PESL_{2}^{\pi ,\theta }\left(x\right)$ depend only on the three expectations $E\left(\theta |x\right)$ , $E\left(\theta^{-1}|x\right)$ , and $E\left(\log\theta |x\right)$ .

### 2.1 Conjugate Prior

The problem of finding the Bayes estimator under a conjugate prior is a standard problem that is treated in almost every text on Mathematical Statistics.

The quantities and expressions of the variance and scale parameters of the normal models (Eqs 1, 2) with a known mean *μ* for the conjugate prior are summarized in Table 1. In the table, *α* > 0 and *β* > 0 are known constants,

$$\alpha^{*}=\alpha +\frac{n}{2},\;\beta^{*}={\left[\frac{1}{\beta }+\frac{1}{2}\overset{n}{\underset{i=1}{\sum }}{\left(x_{i}-\mu \right)}^{2}\right]}^{-1},$$

$$\psi \left(z\right)=\frac{\Gamma^{\prime }\left(z\right)}{\Gamma \left(z\right)}=\frac{d}{dz}\log\Gamma \left(z\right)=digamma\left(z\right)$$

is the digamma function, and $\Gamma \left(z\right)$ is the gamma function. In R software (R Core Team. R, 2021), the function digamma(z) calculates $\psi \left(z\right)$ . The quantities and expressions of the variance parameter *θ* = *σ* ^2^ for the conjugate prior are calculated in and quoted from (Zhang, 2017). The calculations of the quantities and expressions of the scale parameter *θ* = *σ* for the conjugate prior can be found in the Supplementary Material. We remark that the calculations of the quantities and expressions in Table 1 are not trivial, especially $E^{\pi_{c}}\left(\log\theta |x\right)$ .

**TABLE 1:** The quantities and expressions for the conjugate prior.

Quantities	$\begin{array}{c} \text{Expressions for }\theta =\sigma^{2},\\ \text{the variance parameter} \end{array}$	$\begin{array}{c} \text{Expressions for }\theta =\sigma ,\\ \text{the scale parameter} \end{array}$
$\pi_{c}\left(\theta \right)$	$IG\left(\alpha ,\beta \right)$	$SRIG\left(\alpha ,\beta \right)$
$\pi_{c}\left(\theta |x\right)$	$IG\left(\alpha^{*},\beta^{*}\right)$	$SRIG\left(\alpha^{*},\beta^{*}\right)$
$E^{\pi_{c}}\left(\theta |x\right)$	$\frac{1}{\left(\alpha^{*}-1\right)\beta^{*}},\;\text{for }\alpha^{*}>1$	$\frac{\Gamma \left(\alpha^{*}-\frac{1}{2}\right)}{\Gamma \left(\alpha^{*}\right)\beta^{*\frac{1}{2}}}\text{, for }\alpha^{*}>\frac{1}{2}$
$E^{\pi_{c}}\left(\frac{1}{\theta }|x\right)$	α*β*	$\frac{\Gamma \left(\alpha^{*}+\frac{1}{2}\right)\beta^{*\frac{1}{2}}}{\Gamma \left(\alpha^{*}\right)}$
$E^{\pi_{c}}\left(\log\theta |x\right)$	$-\log\beta^{*}-\psi \left(\alpha^{*}\right)$	$-\frac{1}{2}\log\beta^{*}-\frac{1}{2}\psi \left(\alpha^{*}\right)$
$\delta_{s}^{\pi_{c},\theta }\left(x\right)$	$\frac{1}{\alpha^{*}\beta^{*}}$	$\frac{\Gamma \left(\alpha^{*}\right)}{\Gamma \left(\alpha^{*}+\frac{1}{2}\right)\beta^{*\frac{1}{2}}}$
$\delta_{2}^{\pi_{c},\theta }\left(x\right)$	$\frac{1}{\left(\alpha^{*}-1\right)\beta^{*}},\;\text{for }\alpha^{*}>1$	$\frac{\Gamma \left(\alpha^{*}-\frac{1}{2}\right)}{\Gamma \left(\alpha^{*}\right)\beta^{*\frac{1}{2}}}\text{, for }\alpha^{*}>\frac{1}{2}$
$PESL_{s}^{\pi_{c},\theta }\left(x\right)$	$\log\alpha^{*}-\psi \left(\alpha^{*}\right)$	$\log\Gamma \left(\alpha^{*}+\frac{1}{2}\right)-\log\Gamma \left(\alpha^{*}\right)-\frac{1}{2}\psi \left(\alpha^{*}\right)$
$PESL_{2}^{\pi_{c},\theta }\left(x\right)$	$\begin{array}{c} \frac{1}{\alpha^{*}-1}+\log\left(\alpha^{*}-1\right)-\psi \left(\alpha^{*}\right),\\ \text{for }\alpha^{*}>1 \end{array}$	$\begin{array}{c} \frac{\Gamma \left(\alpha^{*}-\frac{1}{2}\right)\Gamma \left(\alpha^{*}+\frac{1}{2}\right)}{\Gamma^{2}\left(\alpha^{*}\right)}-1-\log\Gamma \left(\alpha^{*}-\frac{1}{2}\right)\\ +\log\Gamma \left(\alpha^{*}\right)-\frac{1}{2}\psi \left(\alpha^{*}\right)\text{, for }\alpha^{*}>\frac{1}{2} \end{array}$

### 2.2 Noninformative Priors

Famous noninformative priors include the Jeffreys’s ( (Jeffreys, 1961)), reference ( (Bernardo, 1979; Berger and Bernardo, 1992)), and matching ( (Tibshirani, 1989; Datta and Mukerjee, 2004)) priors. See also (Berger, 2006; Berger et al., 2015) and the references therein.

The Jeffreys’s noninformative prior for *θ* = *σ* ^2^ is

$$\pi_{J}\left(\theta \right)\propto \frac{1}{\theta }\text{ or }\pi_{J}\left(\sigma^{2}\right)\propto \frac{1}{\sigma^{2}}.$$

See Part I (p.66) of (Chen, 2014), where *μ* is assumed known in the normal model $N\left(\mu ,\theta \right)$ . The Jeffreys’s noninformative prior for *θ* = *σ* is

$$\pi_{J}\left(\sigma \right)\propto \frac{1}{\sigma }.$$

See Example 3.5.6 (p.131) of (Robert, 2007), where *μ* is assumed known in the normal model $N\left(\mu ,\sigma^{2}\right)$ .

Since *μ* is assumed known in the normal models, there is only one unknown parameter. Therefore, the reference prior is equal to the Jeffreys’s prior, and the matching prior is also equal to the Jeffreys’s prior (see pp.130–131 of (Ghosh et al., 2006)). In summary, when *μ* is assumed known in the normal models, the three noninformative priors equal, that is,

$$\pi_{n}\left(\theta \right)=\pi_{J}\left(\theta \right)=\pi_{R}\left(\theta \right)=\pi_{M}\left(\theta \right)\propto \frac{1}{\theta }$$

and

$$\pi_{n}\left(\sigma \right)=\pi_{J}\left(\sigma \right)=\pi_{R}\left(\sigma \right)=\pi_{M}\left(\sigma \right)\propto \frac{1}{\sigma },$$

where $\pi_{n}\left(\cdot \right)$ stands for the noninformative prior.

Note that as in many statistics textbooks, the probability density function (pdf) of $\theta ∼IG\left(\alpha ,\beta \right)$ is given by

$$f_{\theta }\left(\theta |\alpha ,\beta \right)=\frac{1}{\Gamma \left(\alpha \right)\beta^{\alpha }}{\left(\frac{1}{\theta }\right)}^{\alpha +1}\exp\left(-\frac{1}{\beta \theta }\right),\;\theta >0,\;\alpha >0,\;\beta >0.$$

The conjugate prior of the scale parameter *θ* = *σ* is a Square Root of the Inverse Gamma (SRIG) distribution that we define below.

DEFINITION 1 *Let* $\theta =\sigma^{2}∼IG\left(\alpha ,\beta \right)$ *with* *α* > 0 *and* *β* > 0*. Then* $\sigma =\sqrt{\theta }∼SRIG\left(\alpha ,\beta \right)$ *and the pdf of* *σ* *is given by*

$$f_{\sigma }\left(\sigma |\alpha ,\beta \right)=\frac{2}{\Gamma \left(\alpha \right)\beta^{\alpha }}{\left(\frac{1}{\sigma }\right)}^{2\alpha +1}\exp\left(-\frac{1}{\beta \sigma^{2}}\right),\;\;\sigma >0,\;\alpha >0,\;\beta >0.$$

Definition 1 gives the definition of the SRIG distribution, which is the conjugate prior of the scale parameter *θ* = *σ* of the normal distribution. Because the SRIG distribution can not be found in standard textbooks, so we give its definition here. Moreover, Definition 1 is reasonable, since

$$\begin{array}{ll} f_{\sigma }\left(\sigma |\alpha ,\beta \right) & =f_{\theta }\left(\theta |\alpha ,\beta \right)\left|\theta^{\prime }\left(\sigma \right)\right|\\ & =\frac{1}{\Gamma \left(\alpha \right)\beta^{\alpha }}{\left(\frac{1}{\sigma^{2}}\right)}^{\alpha +1}\exp\left(-\frac{1}{\beta \sigma^{2}}\right)\cdot 2\sigma \\ & =\frac{2}{\Gamma \left(\alpha \right)\beta^{\alpha }}{\left(\frac{1}{\sigma }\right)}^{2\alpha +1}\exp\left(-\frac{1}{\beta \sigma^{2}}\right). \end{array}$$

We have the following proposition which gives the three expectations of the $SRIG\left(\alpha ,\beta \right)$ distribution. The calculations needed in the proposition can be found in the Supplementary Material. We remark that the calculations of $E\left(\sigma \right)$ and $E\left(\sigma^{-1}\right)$ are straightforward by utilizing a simple transformation of *θ* = *σ* ^2^ and the integration of an $IG\left(\alpha ,\beta \right)$ distribution. However, the calculations of $E\left(\log\sigma \right)$ is skillful by first a transformation of $y=1/\left(\beta \sigma^{2}\right)$ and then a change of the order of integration and differentiation.

PROPOSITION 1 *Let* $\sigma =\sqrt{\theta }∼SRIG\left(\alpha ,\beta \right)$ *with* *α* > 0 *and* *β* > 0*. Then*

$$\begin{array}{ll} E\left(\sigma \right) & =\frac{\Gamma \left(\alpha -\frac{1}{2}\right)}{\Gamma \left(\alpha \right)\beta^{\frac{1}{2}}},for\alpha >\frac{1}{2}\;and\beta >0,\\ E\left(\frac{1}{\sigma }\right) & =\frac{\Gamma \left(\alpha +\frac{1}{2}\right)\beta^{\frac{1}{2}}}{\Gamma \left(\alpha \right)},for\alpha >0\;and\beta >0,\\ E\left(\log\sigma \right) & =-\frac{1}{2}\log\beta -\frac{1}{2}\psi \left(\alpha \right),for\alpha >0\;and\beta >0. \end{array}$$

The relationship between the two distributions $IG\left(\alpha ,\beta \right)$ and $SRIG\left(\alpha ,\beta \right)$ are given in the following proposition whose proof can be found in the Supplementary Material. We remark that the proof of the proposition is straightforward by utilizing monotone transformations *θ* = *σ* ^2^ and $\sigma =\sqrt{\theta }$ .

PROPOSITION 2
$\theta =\sigma^{2}∼IG\left(\alpha ,\beta \right)$ *if and only if* $\sigma =\sqrt{\theta }∼SRIG\left(\alpha ,\beta \right)$ *, where* *α* > 0 *and* *β* > 0*.*

The posterior distributions of *θ* and *σ* for the noninformative priors are given in the following theorem whose proof can be found in the Supplementary Material.

THEOREM 1 *Let* $X|\theta ∼N\left(\mu ,\theta \right)$ *and* $X|\sigma ∼N\left(\mu ,\sigma^{2}\right)$ *where* *μ* *is known and* *θ* = *σ* ^2^ *is unknown,* $\pi \left(\theta \right)\propto \frac{1}{\theta }$ *, and* $\pi \left(\sigma \right)\propto \frac{1}{\sigma }$ *. Then*

$$\pi \left(\theta |x\right)∼IG\left(\tilde{\alpha },\tilde{\beta }\right)\text{ and }\pi \left(\sigma |x\right)∼SRIG\left(\tilde{\alpha },\tilde{\beta }\right),$$

where

$$\tilde{\alpha }=\frac{n}{2}\text{ and }\tilde{\beta }=\frac{2}{\sum_{i=1}^{n}{\left(x_{i}-\mu \right)}^{2}}.$$ (8)

We have the following two remarks for Theorem 1.

Remark 1 Let *θ* = *σ* ^2^. In the derivation of $\pi \left(\sigma |x\right)$ , if we derive it in this way,

$$\begin{array}{ll} f_{\sigma }\left(\sigma \right) & =\pi \left(\sigma |x\right)\\ & \propto {\left(\frac{1}{\sigma }\right)}^{n+1}\exp\left(-\frac{1}{2\sigma^{2}}\overset{n}{\underset{i=1}{\sum }}{\left(x_{i}-\mu \right)}^{2}\right)\\ & ={\left(\frac{1}{\sigma^{2}}\right)}^{\frac{n+1}{2}}\exp\left(-\frac{1}{2\sigma^{2}}\overset{n}{\underset{i=1}{\sum }}{\left(x_{i}-\mu \right)}^{2}\right)\\ & ={\left(\frac{1}{\theta }\right)}^{\frac{n+1}{2}}\exp\left(-\frac{1}{2\theta }\overset{n}{\underset{i=1}{\sum }}{\left(x_{i}-\mu \right)}^{2}\right)=f_{\theta }\left(\theta \right)\\ & ∼IG\left({\tilde{\alpha }}_{1},\tilde{\beta }\right), \end{array}$$

where

$${\tilde{\alpha }}_{1}=\frac{n-1}{2}\text{ and }\tilde{\beta }=\frac{2}{\overset{n}{\underset{i=1}{\sum }}{\left(x_{i}-\mu \right)}^{2}},$$

then by Proposition 2, $f_{\sigma }\left(\sigma \right)=\pi \left(\sigma |x\right)∼SRIG\left({\tilde{\alpha }}_{1},\tilde{\beta }\right)$ , which is different from $SRIG\left(\tilde{\alpha },\tilde{\beta }\right)$ . In fact, the above practice is equivalent to the derivation of the pdf of *θ* in terms of the pdf of *σ* by $f_{\theta }\left(\theta \right)=f_{\sigma }\left(\sigma \right)$ , ignoring the $\left|\sigma^{\prime }\left(\theta \right)\right|$ term, which is obviously wrong. Therefore, the above derivation which is a pitfall for incautious users is wrong. ‖

Remark 2 The two posterior distributions in Theorem 1, $\pi \left(\theta |x\right)∼IG\left(\tilde{\alpha },\tilde{\beta }\right)$ and $\pi \left(\sigma |x\right)∼SRIG\left(\tilde{\alpha },\tilde{\beta }\right)$ , follow Proposition 2 by accident. We have

$$f_{\theta }\left(\theta \right)=\pi \left(\theta |x\right)\propto f\left(x|\theta \right)\pi \left(\theta \right)\propto f\left(x|\theta \right)\frac{1}{\theta }∼IG\left(\tilde{\alpha },\tilde{\beta }\right)$$

and

$$f_{\sigma }\left(\sigma \right)=\pi \left(\sigma |x\right)\propto f\left(x|\sigma \right)\pi \left(\sigma \right)\propto f\left(x|\theta \right)\frac{1}{\sigma }∼SRIG\left(\tilde{\alpha },\tilde{\beta }\right).$$

Note that $\sigma =\sqrt{\theta }$ , and thus

$$f_{\sigma }\left(\sigma \right)\left|\sigma^{\prime }\left(\theta \right)\right|\propto f\left(x|\theta \right)\frac{1}{\sigma }\frac{1}{2\sqrt{\theta }}=f\left(x|\theta \right)\frac{1}{\sqrt{\theta }}\frac{1}{2\sqrt{\theta }}=f\left(x|\theta \right)\frac{1}{2\theta }\propto f_{\theta }\left(\theta \right),$$ (9)

which is the reason why $\pi \left(\theta |x\right)=f_{\theta }\left(\theta \right)$ and $\pi \left(\sigma |x\right)=f_{\sigma }\left(\sigma \right)$ follow Proposition 2. Note that the posterior distributions depend on the prior distributions. If the prior distributions $\pi \left(\theta \right)$ and $\pi \left(\sigma \right)$ are selected different from $\frac{1}{\theta }$ and $\frac{1}{\sigma }$ , then the relationship (Eq. 9) may not be satisfied, and thus $\pi \left(\theta |x\right)$ and $\pi \left(\sigma |x\right)$ may not follow Proposition 2. ‖

#### 2.2.1 The Quantities and Expressions of the Variance Parameter

In this subsubsection, we will calculate the expressions of the quantities (three posterior expectations, two Bayes estimators, and two PESLs) of the variance parameter *θ* = *σ* ^2^.

Now we calculate the three expectations $E\left(\theta |x\right)$ , $E\left(\theta^{-1}|x\right)$ , and $E\left(\log\theta |x\right)$ for the variance parameter *θ* = *σ* ^2^. By Theorem 1, $\pi \left(\theta |x\right)∼IG\left(\tilde{\alpha },\tilde{\beta }\right)$ , and thus

$$E\left(\theta |x\right)=\frac{1}{\left(\tilde{\alpha }-1\right)\tilde{\beta }},\;\tilde{\alpha }>1\text{ and }E\left(\frac{1}{\theta }|x\right)=\tilde{\alpha }\tilde{\beta }.$$

From (Zhang, 2017), we know that

$$E\left(\log\theta |x\right)=-\log\tilde{\beta }-\psi \left(\tilde{\alpha }\right).$$

It is easy to see that, for $\tilde{\alpha }>1$ ,

$$\delta_{s}^{\pi ,\theta }\left(x\right)=\frac{1}{E\left(\frac{1}{\theta }|x\right)}=\frac{1}{\tilde{\alpha }\tilde{\beta }}<\frac{1}{\left(\tilde{\alpha }-1\right)\tilde{\beta }}=E\left(\theta |x\right)=\delta_{2}^{\pi ,\theta }\left(x\right),$$

which exemplifies (Eq. 6). From (Zhang, 2017), we find that

$$PESL_{s}^{\pi ,\theta }\left(x\right)=\log\tilde{\alpha }-\psi \left(\tilde{\alpha }\right),\text{ for }\tilde{\alpha }>0,$$

and

$$PESL_{2}^{\pi ,\theta }\left(x\right)=\frac{1}{\tilde{\alpha }-1}+\log\left(\tilde{\alpha }-1\right)-\psi \left(\tilde{\alpha }\right),\text{ for }\tilde{\alpha }>1.$$

It can be directly proved that $PESL_{s}^{\pi ,\theta }\left(x\right)\leq PESL_{2}^{\pi ,\theta }\left(x\right)$ for $\tilde{\alpha }>1$ , which exemplifies (Eq. 7), and its proof which exploits the Taylor series expansion for *e* ^*x*^ can be found in the Supplementary Material. Note that $PESL_{s}^{\pi ,\theta }\left(x\right)$ and $PESL_{2}^{\pi ,\theta }\left(x\right)$ depend only on $\tilde{\alpha }=n/2$ . Therefore, they depend only on *n*, but do not depend on *μ* and *x*. Numerical simulations will exemplify this result.

The IRSL at $\delta_{s}^{\pi ,\theta }$ or the BRSL for *θ* = *σ* ^2^ is (similar to (Robert, 2007))

$$\begin{array}{ll} & IRSL_{s}^{\pi ,\theta }=BRSL^{\pi ,\theta }=r\left(\pi ,\delta_{s}^{\pi ,\theta }\right)\\ & =E^{\pi }\left[R\left(\theta ,\delta_{s}^{\pi ,\theta }\right)\right]\\ & =\int_{\Theta }R\left(\theta ,\delta_{s}^{\pi ,\theta }\right)\pi \left(\theta \right)d\theta \\ & =\int_{\Theta }\int_{X}L\left(\theta ,\delta_{s}^{\pi ,\theta }\left(x\right)\right)f\left(x|\theta \right)dx\pi \left(\theta \right)d\theta \\ & =\int_{X}\int_{\Theta }L\left(\theta ,\delta_{s}^{\pi ,\theta }\left(x\right)\right)f\left(x|\theta \right)\pi \left(\theta \right)d\theta dx\\ & =\int_{X}\int_{\Theta }L\left(\theta ,\delta_{s}^{\pi ,\theta }\left(x\right)\right)\pi \left(\theta |x\right)d\theta m^{\pi ,\theta }\left(x\right)dx\\ & =\int_{X}{\left.PESL^{\pi ,\theta }\left(a\left(x\right)|x\right)\right|}_{a=\delta_{s}^{\pi ,\theta }}m^{\pi ,\theta }\left(x\right)dx\\ & =\int_{X}PESL_{s}^{\pi ,\theta }\left(x\right)m^{\pi ,\theta }\left(x\right)dx\\ & =\int_{X}\left[\log\tilde{\alpha }-\psi \left(\tilde{\alpha }\right)\right]m^{\pi ,\theta }\left(x\right)dx\\ & =\log\tilde{\alpha }-\psi \left(\tilde{\alpha }\right)\\ & =PESL_{s}^{\pi ,\theta }\left(x\right), \end{array}$$

since $\tilde{\alpha }$ does not depend on *x*, where

$$m^{\pi ,\theta }\left(x\right)=\int_{0}^{\infty }f\left(x|\theta \right)\pi \left(\theta \right)d\theta$$

is the marginal density of **x** with prior $\pi \left(\theta \right)$ .

#### 2.2.2 The Quantities and Expressions of the Scale Parameter

In this subsubsection, we will calculate the expressions of the quantities (three posterior expectations, two Bayes estimators, and two PESLs) of the scale parameter *θ* = *σ*.

Now let us calculate $\delta_{s}^{\pi ,\sigma }\left(x\right)$ , $\delta_{2}^{\pi ,\sigma }\left(x\right)$ , $PESL_{s}^{\pi ,\sigma }\left(x\right)$ , and $PESL_{2}^{\pi ,\sigma }\left(x\right)$ for the scale parameter *σ*. To calculate these quantities, we need to calculate the three expectations $E\left(\sigma |x\right)$ , $E\left(\sigma^{-1}|x\right)$ , and $E\left(\log\sigma |x\right)$ . Since $\pi \left(\sigma |x\right)∼SRIG\left(\tilde{\alpha },\tilde{\beta }\right)$ by Theorem 1, from Proposition 1, we have

$$\begin{array}{ll} E\left(\sigma |x\right) & =\frac{\Gamma \left(\tilde{\alpha }-\frac{1}{2}\right)}{\Gamma \left(\tilde{\alpha }\right){\tilde{\beta }}^{\frac{1}{2}}}\text{, for }\tilde{\alpha }>\frac{1}{2}\text{ and }\tilde{\beta }>0, \end{array}$$ (10)

$$\begin{array}{ll} E\left(\frac{1}{\sigma }|x\right) & =\frac{\Gamma \left(\tilde{\alpha }+\frac{1}{2}\right){\tilde{\beta }}^{\frac{1}{2}}}{\Gamma \left(\tilde{\alpha }\right)}\text{, for }\tilde{\alpha }>0\text{ and }\tilde{\beta }>0, \end{array}$$ (11)

$$\begin{array}{ll} E\left(\log\sigma |x\right) & =-\frac{1}{2}\log\tilde{\beta }-\frac{1}{2}\psi \left(\tilde{\alpha }\right)\text{, for }\tilde{\alpha }>0\text{ and }\tilde{\beta }>0. \end{array}$$ (12)

It can be proved that, for $\tilde{\alpha }>\frac{1}{2}$ ,

$$\delta_{s}^{\pi ,\sigma }\left(x\right)=\frac{1}{E\left(\frac{1}{\sigma }|x\right)}=\frac{\Gamma \left(\tilde{\alpha }\right)}{\Gamma \left(\tilde{\alpha }+\frac{1}{2}\right){\tilde{\beta }}^{\frac{1}{2}}}<\frac{\Gamma \left(\tilde{\alpha }-\frac{1}{2}\right)}{\Gamma \left(\tilde{\alpha }\right){\tilde{\beta }}^{\frac{1}{2}}}=E\left(\sigma |x\right)=\delta_{2}^{\pi ,\sigma }\left(x\right),$$

which exemplifies (Eq. 6), and the proof which exploits the positivity of $\psi^{\prime }\left(x\right)$ can be found in the Supplementary Material.

Now we calculate $PESL_{s}^{\pi ,\sigma }\left(x\right)$ and $PESL_{2}^{\pi ,\sigma }\left(x\right)$ for the scale parameter *σ*. From (Zhang, 2017), we know that the PESL at $\delta_{s}^{\pi ,\sigma }\left(x\right)={\left[E\left(\sigma^{-1}|x\right)\right]}^{-1}$ is

$$PESL_{s}^{\pi ,\sigma }\left(x\right)={\left.E\left[L_{s}\left(\theta ,a\right)|x\right]\right|}_{a=\frac{1}{E\left(\frac{1}{\sigma }|x\right)}}=\log E\left(\frac{1}{\sigma }|x\right)+E\left(\log\sigma |x\right),$$

and the PESL at $\delta_{2}^{\pi ,\sigma }\left(x\right)=E\left(\sigma |x\right)$ is

$$\begin{array}{ll} PESL_{2}^{\pi ,\sigma }\left(x\right) & ={\left.E\left[L_{s}\left(\theta ,a\right)|x\right]\right|}_{a=E\left(\sigma |x\right)}\\ & =E\left(\sigma |x\right)E\left(\frac{1}{\sigma }|x\right)-1-\log E\left(\sigma |x\right)+E\left(\log\sigma |x\right). \end{array}$$

Substituting (Eqs 10, 11, 12), into the above expressions, we obtain

$$\begin{array}{ll} PESL_{s}^{\pi ,\sigma }\left(x\right) & =\log\frac{\Gamma \left(\tilde{\alpha }+\frac{1}{2}\right){\tilde{\beta }}^{\frac{1}{2}}}{\Gamma \left(\tilde{\alpha }\right)}-\frac{1}{2}\log\tilde{\beta }-\frac{1}{2}\psi \left(\tilde{\alpha }\right)\\ & =\log\Gamma \left(\tilde{\alpha }+\frac{1}{2}\right)-\log\Gamma \left(\tilde{\alpha }\right)-\frac{1}{2}\psi \left(\tilde{\alpha }\right), \end{array}$$

for $\tilde{\alpha }>0$ and $\tilde{\beta }>0$ , and

$$\begin{array}{ll} PESL_{2}^{\pi ,\sigma }\left(x\right) & =\frac{\Gamma \left(\tilde{\alpha }-\frac{1}{2}\right)}{\Gamma \left(\tilde{\alpha }\right){\tilde{\beta }}^{\frac{1}{2}}}\frac{\Gamma \left(\tilde{\alpha }+\frac{1}{2}\right){\tilde{\beta }}^{\frac{1}{2}}}{\Gamma \left(\tilde{\alpha }\right)}-1-\log\frac{\Gamma \left(\tilde{\alpha }-\frac{1}{2}\right)}{\Gamma \left(\tilde{\alpha }\right){\tilde{\beta }}^{\frac{1}{2}}}-\frac{1}{2}\log\tilde{\beta }-\frac{1}{2}\psi \left(\tilde{\alpha }\right)\\ & =\frac{\Gamma \left(\tilde{\alpha }-\frac{1}{2}\right)\Gamma \left(\tilde{\alpha }+\frac{1}{2}\right)}{\Gamma^{2}\left(\tilde{\alpha }\right)}-1-\log\Gamma \left(\tilde{\alpha }-\frac{1}{2}\right)+\log\Gamma \left(\tilde{\alpha }\right)-\frac{1}{2}\psi \left(\tilde{\alpha }\right), \end{array}$$

for $\tilde{\alpha }>\frac{1}{2}$ and $\tilde{\beta }>0$ . It can be directly proved that $PESL_{s}^{\pi ,\sigma }\left(x\right)\leq PESL_{2}^{\pi ,\sigma }\left(x\right)$ for $\tilde{\alpha }>\frac{1}{2}$ and $\tilde{\beta }>0$ , which exemplifies (Eq. 7), and its proof which exploits the Taylor series expansion for log  *u* with *u* near 1 can be found in the Supplementary Material. Note that $PESL_{s}^{\pi ,\sigma }\left(x\right)$ and $PESL_{2}^{\pi ,\sigma }\left(x\right)$ depend only on $\tilde{\alpha }=n/2$ . Therefore, they depend only on *n*, but do not depend on *μ* and *x*. Numerical simulations will exemplify this result.

The IRSL at $\delta_{s}^{\pi ,\sigma }$ or the BRSL for *θ* = *σ* is (similar to (Robert, 2007))

$$\begin{array}{ll} & IRSL_{s}^{\pi ,\sigma }=BRSL^{\pi ,\sigma }=r\left(\pi ,\delta_{s}^{\pi ,\sigma }\right)\\ & =E^{\pi }\left[R\left(\sigma ,\delta_{s}^{\pi ,\sigma }\right)\right]\\ & =\int_{\Sigma }R\left(\sigma ,\delta_{s}^{\pi ,\sigma }\right)\pi \left(\sigma \right)d\sigma \\ & =\int_{\Sigma }\int_{X}L\left(\sigma ,\delta_{s}^{\pi ,\sigma }\left(x\right)\right)f\left(x|\sigma \right)dx\pi \left(\sigma \right)d\sigma \\ & =\int_{X}\int_{\Sigma }L\left(\sigma ,\delta_{s}^{\pi ,\sigma }\left(x\right)\right)f\left(x|\sigma \right)\pi \left(\sigma \right)d\sigma dx\\ & =\int_{X}\int_{\Sigma }L\left(\sigma ,\delta_{s}^{\pi ,\sigma }\left(x\right)\right)\pi \left(\sigma |x\right)d\sigma m^{\pi ,\sigma }\left(x\right)dx\\ & =\int_{X}{\left.PESL^{\pi ,\sigma }\left(a\left(x\right)|x\right)\right|}_{a=\delta_{s}^{\pi ,\sigma }}m^{\pi ,\sigma }\left(x\right)dx\\ & =\int_{X}PESL_{s}^{\pi ,\sigma }\left(x\right)m^{\pi ,\sigma }\left(x\right)dx\\ & =\int_{X}\left[\log\Gamma \left(\tilde{\alpha }+\frac{1}{2}\right)-\log\Gamma \left(\tilde{\alpha }\right)-\frac{1}{2}\psi \left(\tilde{\alpha }\right)\right]m^{\pi ,\sigma }\left(x\right)dx\\ & =\log\Gamma \left(\tilde{\alpha }+\frac{1}{2}\right)-\log\Gamma \left(\tilde{\alpha }\right)-\frac{1}{2}\psi \left(\tilde{\alpha }\right)\\ & =PESL_{s}^{\pi ,\sigma }\left(x\right), \end{array}$$

since $\tilde{\alpha }$ does not depend on *x*, where

$$m^{\pi ,\sigma }\left(x\right)=\int_{0}^{\infty }f\left(x|\sigma \right)\pi \left(\sigma \right)d\sigma$$

is the marginal density of **x** with prior $\pi \left(\sigma \right)$ .

The quantities and expressions of the variance and scale parameters for the noninformative priors are summarized in Table 2. In the table, $\tilde{\alpha }$ and $\tilde{\beta }$ are given by (Eq. 8).

**TABLE 2:** The quantities and expressions for the noninformative priors.

Quantities	$\begin{array}{c} \text{Expressions for }\theta =\sigma^{2},\\ \text{the variance parameter} \end{array}$	$\begin{array}{c} \text{Expressions for }\theta =\sigma ,\\ \text{the scale parameter} \end{array}$
$\pi_{n}\left(\theta \right)$	$\frac{1}{\theta }$	$\frac{1}{\sigma }$
$\pi_{n}\left(\theta |x\right)$	$IG\left(\tilde{\alpha },\tilde{\beta }\right)$	$SRIG\left(\tilde{\alpha },\tilde{\beta }\right)$
$E^{\pi_{n}}\left(\theta |x\right)$	$\frac{1}{\left(\tilde{\alpha }-1\right)\tilde{\beta }},\;\text{for }\tilde{\alpha }>1$	$\frac{\Gamma \left(\tilde{\alpha }-\frac{1}{2}\right)}{\Gamma \left(\tilde{\alpha }\right){\tilde{\beta }}^{\frac{1}{2}}}\text{, for }\tilde{\alpha }>\frac{1}{2}$
$E^{\pi_{n}}\left(\frac{1}{\theta }|x\right)$	$\tilde{\alpha }\tilde{\beta }$	$\frac{\Gamma \left(\tilde{\alpha }+\frac{1}{2}\right){\tilde{\beta }}^{\frac{1}{2}}}{\Gamma \left(\tilde{\alpha }\right)}$
$E^{\pi_{n}}\left(\log\theta |x\right)$	$-\log\tilde{\beta }-\psi \left(\tilde{\alpha }\right)$	$-\frac{1}{2}\log\tilde{\beta }-\frac{1}{2}\psi \left(\tilde{\alpha }\right)$
$\delta_{s}^{\pi_{n},\theta }\left(x\right)$	$\frac{1}{\tilde{\alpha }\tilde{\beta }}$	$\frac{\Gamma \left(\tilde{\alpha }\right)}{\Gamma \left(\tilde{\alpha }+\frac{1}{2}\right){\tilde{\beta }}^{\frac{1}{2}}}$
$\delta_{2}^{\pi_{n},\theta }\left(x\right)$	$\frac{1}{\left(\tilde{\alpha }-1\right)\tilde{\beta }},\;\text{for }\tilde{\alpha }>1$	$\frac{\Gamma \left(\tilde{\alpha }-\frac{1}{2}\right)}{\Gamma \left(\tilde{\alpha }\right){\tilde{\beta }}^{\frac{1}{2}}}\text{, for }\tilde{\alpha }>\frac{1}{2}$
$PESL_{s}^{\pi_{n},\theta }\left(x\right)$	$\log\tilde{\alpha }-\psi \left(\tilde{\alpha }\right)$	$\log\Gamma \left(\tilde{\alpha }+\frac{1}{2}\right)-\log\Gamma \left(\tilde{\alpha }\right)-\frac{1}{2}\psi \left(\tilde{\alpha }\right)$
$PESL_{2}^{\pi_{n},\theta }\left(x\right)$	$\begin{array}{c} \frac{1}{\tilde{\alpha }-1}+\log\left(\tilde{\alpha }-1\right)-\psi \left(\tilde{\alpha }\right),\\ \text{for }\tilde{\alpha }>1 \end{array}$	$\begin{array}{c} \frac{\Gamma \left(\tilde{\alpha }-\frac{1}{2}\right)\Gamma \left(\tilde{\alpha }+\frac{1}{2}\right)}{\Gamma^{2}\left(\tilde{\alpha }\right)}-1-\log\Gamma \left(\tilde{\alpha }-\frac{1}{2}\right)\\ +\log\Gamma \left(\tilde{\alpha }\right)-\frac{1}{2}\psi \left(\tilde{\alpha }\right)\text{, for }\tilde{\alpha }>\frac{1}{2} \end{array}$

From Tables 1, 2, we find that there are four combinations of the expressions of the quantities: conjugate prior and variance parameter, conjugate prior and scale parameter, noninformative prior and variance parameter, and noninformative prior and scale parameter. The forms of the expressions of the quantities are the same for the variance parameter under the conjugate and noninformative priors, since they have the same Inverse Gamma posterior distributions. Similarly, the forms of the expressions of the quantities are the same for the scale parameter under the conjugate and noninformative priors, since they have the same Square Root of the Inverse Gamma posterior distributions.

The inequalities (Eqs 6, 7) exist in Tables 1, 2. In fact, there are 8 inequalities in Tables 1, 2 and 4 inequalities in each table. Since the forms of the expressions of the quantities are the same in Tables 1, 2, with the only difference of the parameters, there are actually 4 different inequalities which are in Table 2. One inequality of the four inequalities about the Bayes estimators is obvious, and the proofs of the other three inequalities can be found in the Supplementary Material.

## 3 Numerical Simulations

In this section, we will numerically exemplify the theoretical studies of (Eqs 6, 7), and that the PESLs depend only on *n*, but do not depend on *μ* and *x*. The numerical simulation results are similar for the four combinations of the expressions of the quantities, and thus we only present the results for the combination of the noninformative prior and the scale parameter.

First, we fix *μ* = 0 and *n* = 10, and assume that *σ* = 1 is drawn from the improper prior distribution. After that, we draw a random sample

x=rnorm(n = n, mean = μ, sd = σ)

from *N*(*μ*, *σ* ^2^).

To generate a random sample $\sigma =\left(\sigma_{1},\ldots ,\sigma_{k}\right)$ with *k* = 1000 from

$$\pi_{n}\left(\sigma |x\right)=SRIG\left(\tilde{\alpha },\tilde{\beta }\right),$$

we will adopt the following algorithm. First, compute $\tilde{\alpha }$ and $\tilde{\beta }$ from (Eq. 8). Second, generate a random sample

$$G=\text{rgamma}\left(\text{n }=\;k\text{, shape }=\;\tilde{\alpha }\text{, scale }=\;\tilde{\beta }\right)∼G\left(\tilde{\alpha },\tilde{\beta }\right).$$

Third, compute

$$IG=\frac{1}{G}∼IG\left(\tilde{\alpha },\tilde{\beta }\right).$$

Fourth, compute

$$\sigma =\sqrt{IG}∼SRIG\left(\tilde{\alpha },\tilde{\beta }\right).$$

**FIGURE 2:**
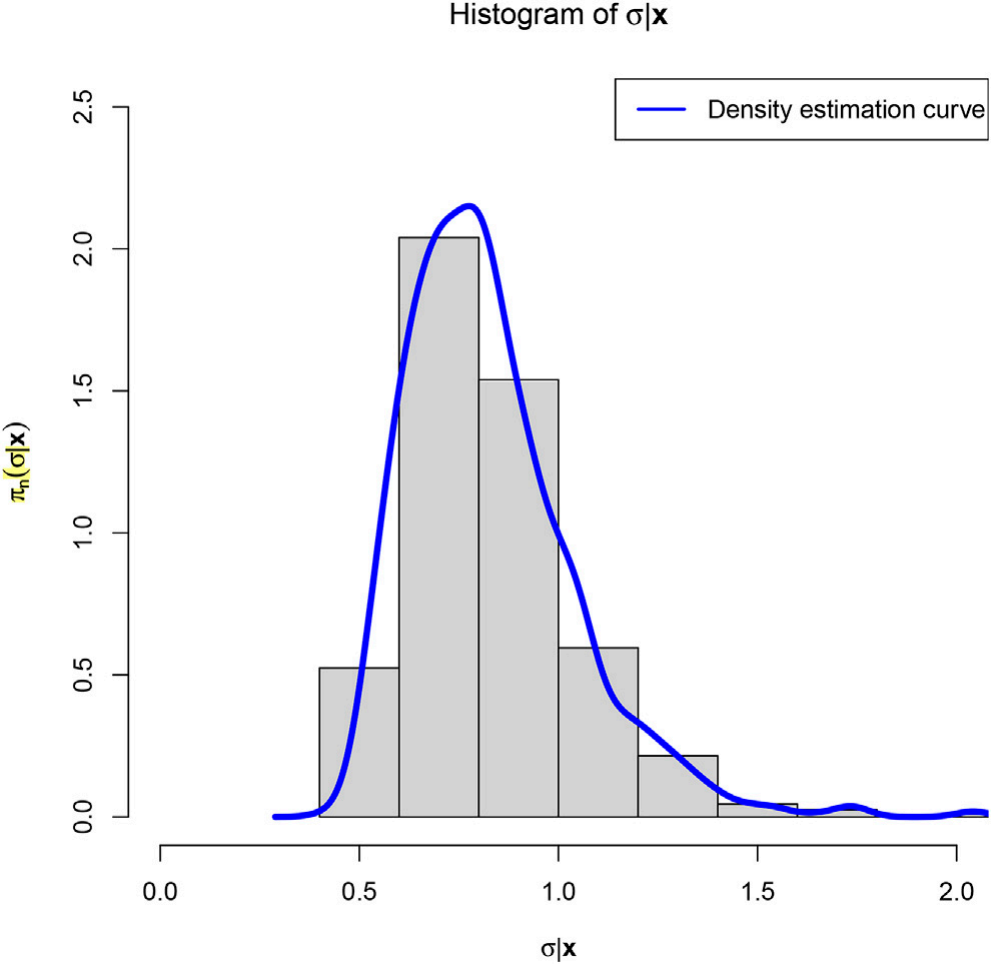
The histogram of *σ*|*x* and the density estimation curve of *π*_*n*_(*σ*|*x*).

Hence, *σ* is a random sample from the $SRIG\left(\tilde{\alpha },\tilde{\beta }\right)$ distribution. Figure 2 shows the histogram of *σ*|*x* and the density estimation curve of *π*_*n*_(*σ*|*x*). It is *π*_*n*_(*σ*|*x*) that we find $\delta_{s}^{\pi_{n},\sigma }\left(x\right)$ to minimize the PESL. From the figure, we see that the $SRIG\left(\tilde{\alpha },\tilde{\beta }\right)$ distribution is left peaked, right skewed, and continuous.

The Bayes estimators ( $\delta_{s}^{\pi_{n},\sigma }\left(x\right)$ and $\delta_{2}^{\pi_{n},\sigma }\left(x\right)$ ) and the PESLs ( $PESL_{s}^{\pi_{n},\sigma }\left(x\right)$ and $PESL_{2}^{\pi_{n},\sigma }\left(x\right)$ ) are computed by the following algorithm. First, compute $\tilde{\alpha }$ and $\tilde{\beta }$ from (Eq. 8). Second, compute

$$\begin{array}{ll} E_{1} & =E\left(\sigma |x\right)=\frac{\Gamma \left(\tilde{\alpha }-\frac{1}{2}\right)}{\Gamma \left(\tilde{\alpha }\right){\tilde{\beta }}^{\frac{1}{2}}}\text{,}\\ E_{2} & =E\left(\frac{1}{\sigma }|x\right)=\frac{\Gamma \left(\tilde{\alpha }+\frac{1}{2}\right){\tilde{\beta }}^{\frac{1}{2}}}{\Gamma \left(\tilde{\alpha }\right)}\text{,}\\ E_{3} & =E\left(\log\sigma |x\right)=-\frac{1}{2}\log\tilde{\beta }-\frac{1}{2}\psi \left(\tilde{\alpha }\right)\text{.} \end{array}$$

Third, compute

$$\begin{array}{ll} \delta_{s}^{\pi_{n},\sigma }\left(x\right) & =\frac{1}{E_{2}},\\ \delta_{2}^{\pi_{n},\sigma }\left(x\right) & =E_{1},\\ PESL_{s}^{\pi_{n},\sigma }\left(x\right) & =\log\left(E_{2}\right)+E_{3},\\ PESL_{2}^{\pi_{n},\sigma }\left(x\right) & =E_{1}\times E_{2}-\log\left(E_{1}\right)+E_{3}-1. \end{array}$$

Numerical results show that

$$\delta_{s}^{\pi_{n},\sigma }\left(x\right)=0.7712483<0.8152161=\delta_{2}^{\pi_{n},\sigma }\left(x\right)$$

and

$$PESL_{s}^{\pi_{n},\sigma }\left(x\right)=0.0267013<0.02826706=PESL_{2}^{\pi_{n},\sigma }\left(x\right),$$

which exemplify the theoretical studies of (6) and (7).

**FIGURE 3:**
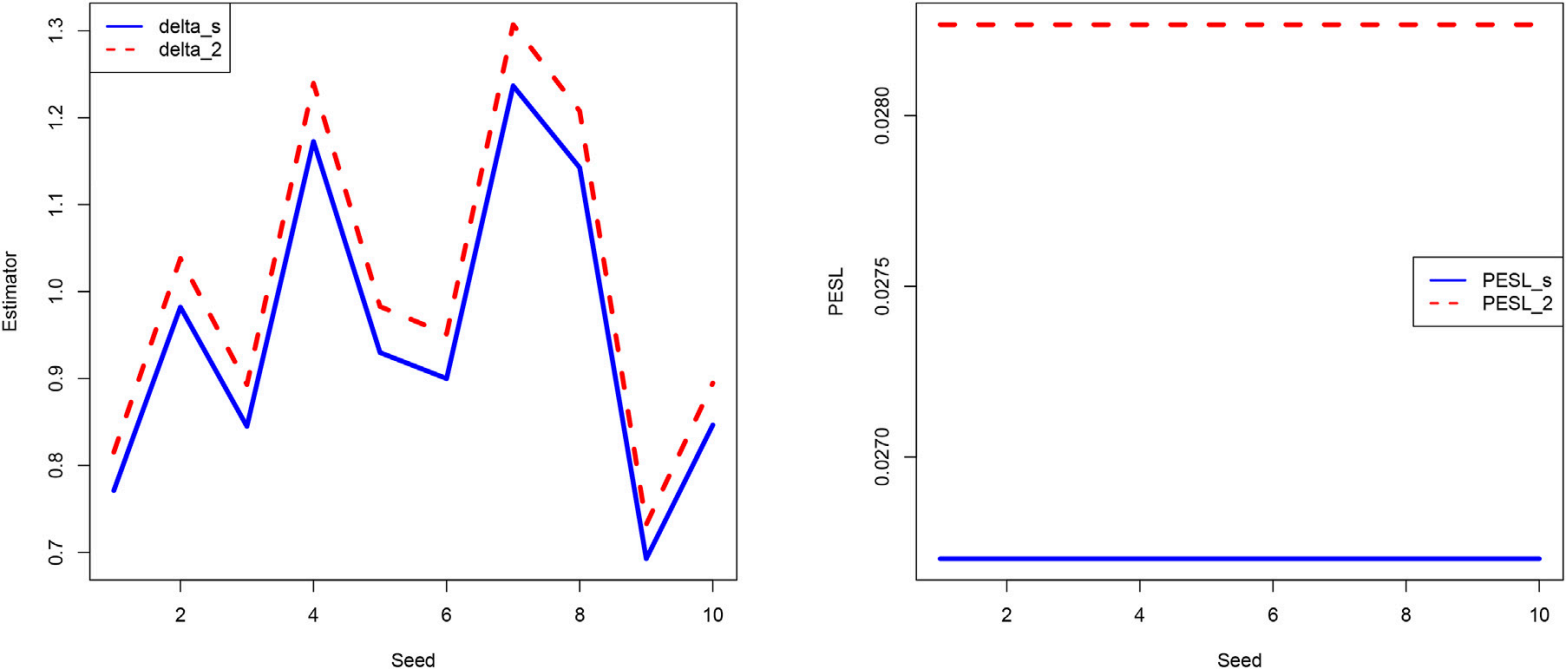
The estimators are functions of *x* **(left)** and the PESLs are also functions of *x* **(right)**.

In Figure 3, we fix *μ* = 0 and *n* = 10, but allow the seed number to change from 1 to 10 (i.e., we change *x*). From the figure we see that the estimators and PESLs are functions of *x*. We see from the left plot of the figure that the estimators depend on *x* in an unpredictable manner, and $\delta_{s}^{\pi_{n},\sigma }\left(x\right)$ are unanimously smaller than $\delta_{2}^{\pi_{n},\sigma }\left(x\right)$ , and thus (Eq. 6) is exemplified. The two Bayes estimators are distinguishable since we fix *n* = 10 to be a small number. The right plot of the figure exhibits that the PESLs do not depend on *x*, and $PESL_{s}^{\pi_{n},\sigma }\left(x\right)$ are unanimously smaller than $PESL_{2}^{\pi_{n},\sigma }\left(x\right)$ , and thus (Eq. 7) is exemplified.

**FIGURE 4:**
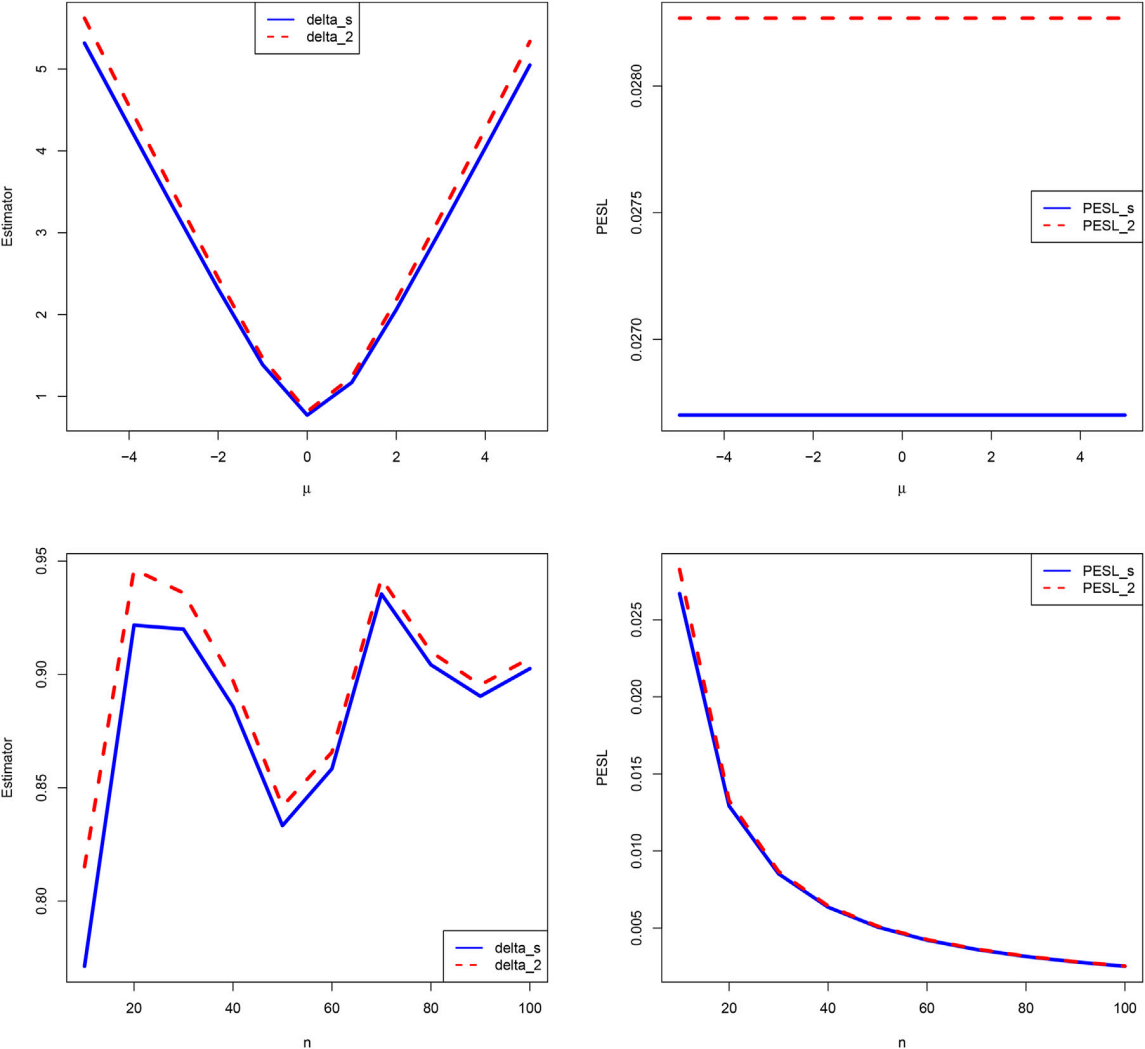
Left: The estimators as functions of *μ* and *n*. Right: The PESLs as functions of *μ* and *n*.

Now we allow one of the two parameters *μ* and *n* to change, holding other parameters fixed. Moreover, we also assume that the sample *x* is fixed, as it is the case for the real data. Figure 4 shows the estimators and PESLs as functions of *μ* and *n*. We see from the left plots of the figure that the estimators depend on *μ* and *n*, and (Eq. 6) is exemplified. More specifically, the estimators are first decreasing and then increasing functions of *μ*, and the estimators attain the minimum when *μ* = 0. However, the estimators fluctuate around some value when *n* increases. The right plots of the figure exhibit that the PESLs depend only on *n*, but do not depend on *μ* , and (Eq. 7) is exemplified. More specifically, the PESLs are decreasing functions of *n*. Furthermore, the two PESLs as functions of *n* are indistinguishable, as the two PESLs are very close. In summary, the results of the figure exemplify the theoretical studies of (Eqs 6, 7).

**FIGURE 5:**
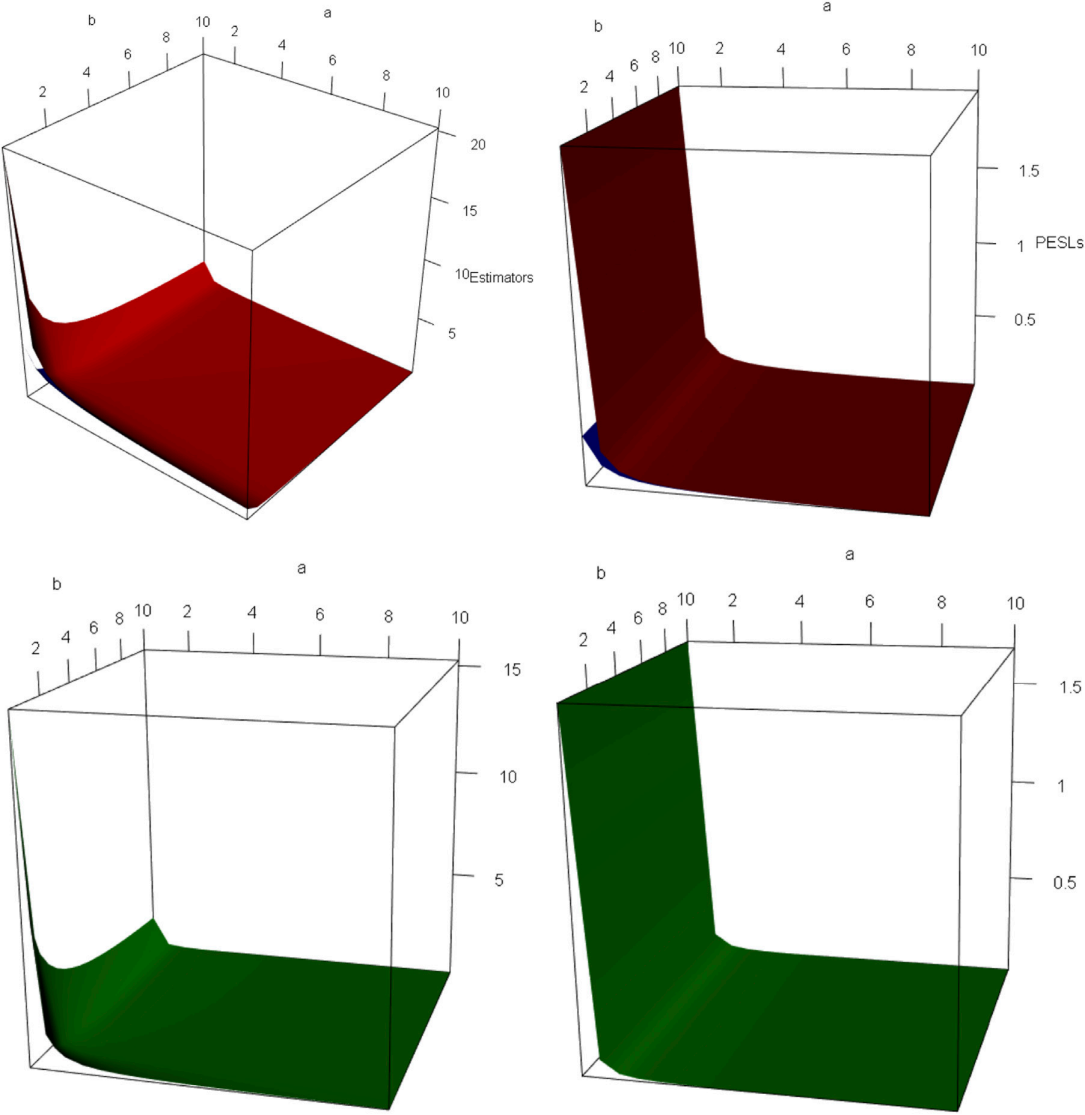
The domain for $\left(\tilde{\alpha },\tilde{\beta }\right)$ is *D* = (0.5, 10] × (0, 10] for all the plots. a is for $\tilde{\alpha }$ and b is for $\tilde{\beta }$ in the axes of all the plots. The red surface is for $\delta_{2}^{\pi_{n},\sigma }\left(x\right)$ and the blue surface is for $\delta_{s}^{\pi_{n},\sigma }\left(x\right)$ in the upper two plots. **(upper left)** The estimators as functions of $\tilde{\alpha }$ and $\tilde{\beta }$ . $\delta_{s}^{\pi_{n},\sigma }\left(x\right)<\delta_{2}^{\pi_{n},\sigma }\left(x\right)$ for all $\left(\tilde{\alpha },\tilde{\beta }\right)$ on *D*. **(upper right)** The PESLs as functions of $\tilde{\alpha }$ and $\tilde{\beta }$ . $PESL_{s}^{\pi_{n},\sigma }\left(x\right)<PESL_{2}^{\pi_{n},\sigma }\left(x\right)$ for all $\left(\tilde{\alpha },\tilde{\beta }\right)$ on *D*. **(lower left)** The surface of $\delta_{2}^{\pi_{n},\sigma }\left(x\right)-\delta_{s}^{\pi_{n},\sigma }\left(x\right)$ which is positive for all $\left(\tilde{\alpha },\tilde{\beta }\right)$ on *D*. **(lower right)** The surface of $PESL_{2}^{\pi_{n},\sigma }\left(x\right)-PESL_{s}^{\pi_{n},\sigma }\left(x\right)$ which is also positive for all $\left(\tilde{\alpha },\tilde{\beta }\right)$ on *D*.

Since the estimators $\delta_{s}^{\pi_{n},\sigma }\left(x\right)$ and $\delta_{2}^{\pi_{n},\sigma }\left(x\right)$ and the PESLs $PESL_{s}^{\pi_{n},\sigma }\left(x\right)$ and $PESL_{2}^{\pi_{n},\sigma }\left(x\right)$ depend on $\tilde{\alpha }$ and $\tilde{\beta }$ , where $\tilde{\alpha }>1/2$ and $\tilde{\beta }>0$ , we can plot the surfaces of the estimators and the PESLs on the domain $\left(\tilde{\alpha },\tilde{\beta }\right)\in \left(0.5,10\right]\times \left(0,10\right]=D$ via the R function persp3d() in the R package rgl (see (Adler and Murdoch, 2017; Zhang et al., 2017; Zhang et al., 2019; Sun et al., 2021)). We remark that the R function persp() in the R package graphics can not add another surface to the existing surface, but persp3d() can. Moreover, persp3d() allows one to rotate the perspective plots of the surface according to one’s wishes. Figure 5 plots the surfaces of the estimators and the PESLs, and the surfaces of the difference of the estimators and the difference of the PESLs. From the left two plots of the figure, we see that $\delta_{s}^{\pi_{n},\sigma }\left(x\right)<\delta_{2}^{\pi_{n},\sigma }\left(x\right)$ for all $\left(\tilde{\alpha },\tilde{\beta }\right)$ on *D*, which exemplifies (Eq. 6). From the right two plots of the figure, we see that $PESL_{s}^{\pi_{n},\sigma }\left(x\right)<PESL_{2}^{\pi_{n},\sigma }\left(x\right)$ for all $\left(\tilde{\alpha },\tilde{\beta }\right)$ on *D*, which exemplifies (Eq. 7). In summary, the results of the figure exemplify the theoretical studies of (Eqs 6, 7).

## 4 A Real Data Example

In this section, we exploit the data from finance. The R package quantmod ( (Ryan and Ulrich, 2017)) is exploited to download the data ˆGSPC (the S&P 500) during 2020-04-24 and 2021-07-02 from “finance.yahoo.com.” It is commonly believed that the monthly simple returns of the index data or the stock data are normally distributed. It is simple to check that the S&P 500 monthly simple returns follow the normal model. Usually, the data from real examples can be regarded as iid from the normal model with an unknown mean *μ*. However, the mean *μ* could be estimated by prior information or historical information. Alternatively, the mean *μ* could be estimated by the sample mean. Therefore, for simplicity, we assume that the mean *μ* is known. Assume that

$$\mu =\bar{x},\;\alpha =1,\;\beta =1$$

for the S&P 500 monthly simple returns.

The Bayes estimators and the PESLs of the variance and scale parameters of the S&P 500 monthly simple returns for the conjugate and noninformative priors are summarized in Table 3. From the table, we observe the following facts.

• The two inequalities (Eqs 6, 7) are exemplified.
• Given the prior (conjugate or noninformative), the Bayes estimators are similar across different loss functions (Stein’s or squared error).
• Given the loss function, the Bayes estimators are quite different across different priors. Therefore, the prior has a larger influence than the loss function in calculating the Bayes estimators.

**TABLE 3:** The Bayes estimators and the PESLs of the S&P 500 monthly simple returns.

	Conjugate prior		Noninformative prior	
	*θ* = *σ* ^2^	*θ* = *σ*	*θ* = *σ* ^2^	*θ* = *σ*
$\delta_{s}^{\pi ,\theta }\left(x\right)$	0.111474	0.338545	0.000408	0.020528
$\delta_{2}^{\pi ,\theta }\left(x\right)$	0.125408	0.348644	0.000467	0.021224
$PESL_{s}^{\pi ,\theta }\left(x\right)$	0.056583	0.014410	0.063800	0.016285
$PESL_{2}^{\pi ,\theta }\left(x\right)$	0.063800	0.014846	0.073126	0.016846

More results (the data of the S&P 500 monthly simple returns, the plot of the S&P 500 monthly close prices, the plot of the S&P 500 monthly simple returns, the histogram of the S&P 500 monthly simple returns) for the real data example can be found in the Supplementary Material due to space limitations.

## 5 Conclusions and Discussions

For the variance (*θ* = *σ* ^2^) and scale (*θ* = *σ*) parameters of the normal model with a known mean *μ*, we recommend and analytically calculate the Bayes estimators, $\delta_{s}^{\pi ,\theta }\left(x\right)$ , with respect to the conjugate and noninformative (Jeffreys’s, reference, and matching) priors under Stein’s loss function which penalizes gross overestimation and gross underestimation equally. These estimators minimize the PESLs. We also analytically calculate the Bayes estimators, $\delta_{2}^{\pi ,\theta }\left(x\right)=E\left(\theta |x\right)$ , with respect to the conjugate and noninformative priors under the squared error loss function, and the corresponding PESLs. The quantities ( $\pi \left(\theta \right)$ , $\pi \left(\theta |x\right)$ , $E^{\pi }\left(\theta |x\right)$ , $E^{\pi }\left(\theta^{-1}|x\right)$ , $E^{\pi }\left(\log\theta |x\right)$ , $\delta_{s}^{\pi ,\theta }\left(x\right)$ , $\delta_{2}^{\pi ,\theta }\left(x\right)$ , $PESL_{s}^{\pi ,\theta }\left(x\right)$ , $PESL_{2}^{\pi ,\theta }\left(x\right)$ ) and expressions of the variance and scale parameters for the conjugate and noninformative priors are summarized in Tables 1, 2, respectively. Note that $E^{\pi }\left(\log\theta |x\right)$ , which is essential for the calculation of $PESL_{s}^{\pi ,\theta }\left(x\right)$ and $PESL_{2}^{\pi ,\theta }\left(x\right)$ , depends on the digamma function.

Proposition 1 gives the three expectations of the $SRIG\left(\alpha ,\beta \right)$ distribution. Moreover, Proposition 2 gives the relationship between the two distributions $IG\left(\alpha ,\beta \right)$ and $SRIG\left(\alpha ,\beta \right)$ .

For the conjugate and noninformative priors, the posterior distribution of *θ* = *σ* ^2^, $\pi \left(\theta |x\right)$ , follows an Inverse Gamma distribution, and the posterior distribution of *σ*, $\pi \left(\sigma |x\right)$ , follows an SRIG distribution which is defined in Definition 1.

We find that the IRSL at $\delta_{s}^{\pi ,\theta }$ or the BRSL for *θ* = *σ* ^2^ is

$$PESL_{s}^{\pi ,\theta }\left(x\right)=\log\tilde{\alpha }-\psi \left(\tilde{\alpha }\right).$$

In addition, the IRSL at $\delta_{s}^{\pi ,\sigma }$ or the BRSL for *θ* = *σ* is

$$PESL_{s}^{\pi ,\sigma }\left(x\right)=\log\Gamma \left(\tilde{\alpha }+\frac{1}{2}\right)-\log\Gamma \left(\tilde{\alpha }\right)-\frac{1}{2}\psi \left(\tilde{\alpha }\right).$$

The numerical simulations of the combination of the noninformative prior and the scale parameter exemplify the theoretical studies of (Eqs 6, 7), and that the PESLs depend only on *n*, but do not depend on *μ* and *x*. Moreover, in the real data example, we have calculated the Bayes estimators and the PESLs of the variance and scale parameters of the S&P 500 monthly simple returns for the conjugate and noninformative priors.

Unlike in frequentist paradigm, if $\hat{\sigma }$ is the Maximum Likelihood Estimator (MLE) of *σ*, then ${\hat{\sigma }}^{2}$ is the MLE of *σ* ^2^. In Bayesian paradigm, we usually should estimate the variance parameter *σ* ^2^ and the scale parameter *σ* separately. In Table 2, we find that

$$\delta_{s}^{\pi_{n},\sigma^{2}}\left(x\right)=\frac{1}{\tilde{\alpha }\tilde{\beta }}\text{ and }\delta_{s}^{\pi_{n},\sigma }\left(x\right)=\frac{\Gamma \left(\tilde{\alpha }\right)}{\Gamma \left(\tilde{\alpha }+\frac{1}{2}\right){\tilde{\beta }}^{\frac{1}{2}}}.$$

It is easy to see that

$$\delta_{s}^{\pi_{n},\sigma^{2}}\left(x\right)\neq {\left[\delta_{s}^{\pi_{n},\sigma }\left(x\right)\right]}^{2}.$$

Similarly,

$$\delta_{2}^{\pi_{n},\sigma^{2}}\left(x\right)\neq {\left[\delta_{2}^{\pi_{n},\sigma }\left(x\right)\right]}^{2}.$$

When there is no prior information about the unknown parameter of interest, we prefer the noninformative prior, as the hyperparameters *α* and *β* are somewhat arbitrary for the conjugate prior.

We remark that the Bayes estimator under Stein’s loss function is more appropriate than that under the squared error loss function, not because the former is smaller, but because Stein’s loss function which penalizes gross overestimation and gross underestimation equally is more appropriate for the positive restricted parameter.

## Data Availability

The original contributions presented in the study are included in the article/Supplementary Material, further inquiries can be directed to the corresponding author.
