# Supplementary material for: The Bayes Estimators of the Variance and Scale Parameters of the Normal Model With a Known Mean for the Conjugate and Noninformative Priors Under Stein’s Loss
Source: Front Big Data. 2022 Jan 3;4:763925. doi: 10.3389/fdata.2021.763925 (PMC8763389; doi:10.3389/fdata.2021.763925)
Supplement: Supplementary file 1 [file DataSheet2.PDF]

Appendix

This is the appendix of the paper. In the appendix, we provide the correct (error free) code for graphs and for all numerical values so that it becomes attractive and practicable for the readers to replicate the results.

A.1. MAIN_LOSS_FUNCTIONS.R

```
####################################################
### code chunk number : set your work directory
####################################################
##
## Firstly, set your work directory to contain "main_loss_functions.R".
## Secondly, make sure there is a folder named "figure"
## in the same directory of "main_loss_functions.R".
##


####################################################
### code chunk number : Compare loss functions L(theta, a), theta = 2
####################################################
##
## Compare loss functions:
## L_2(theta, a), L_s(theta, a), L_L(theta, a | c = 1), and L_L(theta, a | c = -1)
##
rm(list=ls(all=TRUE))

theta = 2
a = c(seq(1e-10, 2, by = 0.001), seq(2, 60, by = 10))
y1 = (a - theta)^2 # L_2(theta, a)
y2 = a / theta - log(a / theta) - 1 # L_s(theta, a)
c = 1
y3 = exp(c * (a - theta)) - c * (a - theta) - 1 # L_L(theta, a | c = 1)
c = -1
y4 = exp(c * (a - theta)) - c * (a - theta) - 1 # L_L(theta, a | c = -1)

dev.new()
col = c("black", "red", "blue", "purple")
lty = 1:4
ylim = c(0, 30) ## range(y1, y2)
plot(a, y1, type = "n", ylim = ylim, xlab = "a", ylab = expression( L(theta, a) ))
lines(a, y1, lty = lty[1], col = col[1], lwd = 4)
lines(a, y2, lty = lty[2], col = col[2], lwd = 4)
lines(a, y3, lty = lty[3], col = col[3], lwd = 4)
lines(a, y4, lty = lty[4], col = col[4], lwd = 4)
```

```
leg.txt = c(expression( L[2](theta, a) ),
expression( L[s](theta, a) ),
expression(paste(L[L], "(", theta, ", ", a, " | c = 1)", sep = "")),
expression(paste(L[L], "(", theta, ", ", a, " | c = -1)", sep = "")))
legend("bottomright", legend = leg.txt, lty = lty, col = col, lwd = 2)
savePlot(filename = "figure/L_2_L_s_L_LINEX", type = c("pdf"))
savePlot(filename = "figure/L_2_L_s_L_LINEX", type = c("jpg"))
```

A.2. MAIN_NONINFORMATIVE_SCALE.R

```
####################################################
####################################################
### noninformative prior, scale parameter
####################################################
####################################################


####################################################
### code chunk number : set your work directory
####################################################
##
## Firstly, set your work directory to contain "main_noninformative_scale.R".
## Secondly, make sure there is a folder named "figure"
## in the same directory of "main_noninformative_scale.R".
##


####################################################
### code chunk number : Given (mu, n, x)
####################################################
##
rm(list=ls(all=TRUE))
source("subfunctions.R")
mu = 0
n = 10

set.seed(1)
sigma = 1 ## assume
x = rnorm(n, mean = mu, sd = sigma); x
res = Compute_PostDist_Estimators_PESLs_noninformative_scale(mu, n, x); res

dev.new()
xlim = c(0, 2)
ylim = c(0, 2.5)
hist(res$PostDist_theta, freq = FALSE,
```

```
     xlab = expression(paste(sigma, "|", bold(x))),
     ylab = expression( pi[n](paste(sigma, "|", bold(x)))),
     xlim = xlim, ylim = ylim,
     main = expression(paste("Histogram of ", sigma, "|", bold(x))) )
lines(density(res$PostDist_theta), lty = 1, col = c('blue'), lwd = 4)
leg.txt = c("Density estimation curve")
legend("topright", legend = leg.txt, lty = 1, col = c('blue'), lwd = 2)
savePlot(filename = "figure/noninformative_scale_density_estimation_curve",
         type = c("pdf"))
savePlot(filename = "figure/noninformative_scale_density_estimation_curve",
         type = c("jpg"))


###################################################
### code chunk number : x change
###################################################
##
## Given (mu, n), x change
##
rm(list=ls(all=TRUE))
source("subfunctions.R")
mu = 0
n = 10
Seed = 1:10 ## x change
L_x = length(Seed)

delta_s = delta_2 = PESL_s = PESL_2 = rep(0, L_x)
for (i in 1:L_x){
     set.seed(i)
     sigma_i = 1 ## assume
     x_i = rnorm(n, mean = mu, sd = sigma_i)
     res_i = Compute_PostDist_Estimators_PESLs_noninformative_scale(mu, n, x_i)
     delta_s[i] = res_i$delta_s
     delta_2[i] = res_i$delta_2
     PESL_s[i] = res_i$PESL_s
     PESL_2[i] = res_i$PESL_2
}

plot_estimator(delta_s, delta_2, parameter = Seed,
     para_name = "noninformative_scale_Seed", legend_pos = "topleft")
plot_PESL(PESL_s, PESL_2, parameter = Seed,
     para_name = "noninformative_scale_Seed", legend_pos = "right")
```

```
####################################################
### code chunk number : mu change
####################################################
##
## Given n, mu change
##
rm(list=ls(all=TRUE))
source("subfunctions.R")
n = 10

mu = -5:5
L_mu = length(mu)

set.seed(1)
x = rnorm(n, mean = 0, sd = 1)

delta_s = delta_2 = PESL_s = PESL_2 = rep(0, L_mu)
for (i in 1:L_mu){
     res_i = Compute_PostDist_Estimators_PESLs_noninformative_scale(mu[i], n, x)
     delta_s[i] = res_i$delta_s
     delta_2[i] = res_i$delta_2
     PESL_s[i] = res_i$PESL_s
     PESL_2[i] = res_i$PESL_2
}

plot_estimator(delta_s, delta_2, parameter = mu,
     para_name = "noninformative_scale_mu", legend_pos = "top")
plot_PESL(PESL_s, PESL_2, parameter = mu,
     para_name = "noninformative_scale_mu", legend_pos = "right")


####################################################
### code chunk number : n change
####################################################
##
## Given mu, n change
##
rm(list=ls(all=TRUE))
source("subfunctions.R")
mu = 0

n = seq(10, 100, by = 10)
L_n = length(n)
```

```
set.seed(1)
x = rnorm(n[L_n], mean = mu, sd = 1)

delta_s = delta_2 = PESL_s = PESL_2 = rep(0, L_n)
for (i in 1:L_n){
     res_i = Compute_PostDist_Estimators_PESLs_noninformative_scale(
             mu, n[i], x[1:n[i]])
     delta_s[i] = res_i$delta_s
     delta_2[i] = res_i$delta_2
     PESL_s[i] = res_i$PESL_s
     PESL_2[i] = res_i$PESL_2
}

plot_estimator(delta_s, delta_2, parameter = n,
     para_name = "noninformative_scale_n", legend_pos = "bottomright")
plot_PESL(PESL_s, PESL_2, parameter = n,
     para_name = "noninformative_scale_n", legend_pos = "topright")


###################################################
### code chunk number : persp3d
###################################################
##
## delta_s <= delta_2
## PESL_s <= PESL_2
##

## Install the package for the first time.
## note: omit the 's' in 'https' if you cannot handle https downloads
## install.packages(pkgs = "rgl", repos = "https://cloud.r-project.org/",
##                  dependencies=TRUE)

rm(list=ls(all=TRUE))
library(rgl) ## Install the R package rgl first.
a = seq(0.6, 10, length = 20); L_a = length(a); L_a ## a > 1/2
b = seq(0.1, 10, length = 20); L_b = length(b); L_b

E1 = function(a, b){ gamma(a - 1/2) / (gamma(a) * sqrt(b)) } ## a > 1/2
E2 = function(a, b){ gamma(a + 1/2) * sqrt(b) / gamma(a) }
E3 = function(a, b){ - log(b)/2 - digamma(a)/2 }

delta_s = function(a, b){ 1 / E2(a, b) }
delta_2 = function(a, b){ E1(a, b) }
PESL_s = function(a, b){ log(E2(a, b)) + E3(a, b) }
PESL_2 = function(a, b){ E1(a, b) * E2(a, b) - log(E1(a, b)) + E3(a, b) - 1 }
```

```
delta_2_s = function(a, b){ delta_2(a, b) - delta_s(a, b) }
PESL_2_s  = function(a, b){ PESL_2(a, b) - PESL_s(a, b) }

## Estimators
z_delta_2 = outer(a, b, FUN = "delta_2")
range(z_delta_2)
dim(z_delta_2)

z_delta_s = outer(a, b, FUN = "delta_s")
range(z_delta_s)
dim(z_delta_s)

range(z_delta_2 - z_delta_s)

z_delta_2_s = outer(a, b, FUN = "delta_2_s")
range(z_delta_2_s)

open3d()
persp3d(a, b, z_delta_2, col = "red", xlab = "a", ylab = "b", zlab = "Estimators")
persp3d(a, b, z_delta_s, col = "blue", add = TRUE)
rgl.postscript("figure/noninformative_scale_Estimators.pdf", "pdf")

open3d()
persp3d(a, b, z_delta_2_s, col = "green", xlab = "a", ylab = "b", zlab = "")
rgl.postscript("figure/noninformative_scale_delta_2_s.pdf", "pdf")

## PESLs
z_PESL_2 = outer(a, b, FUN = "PESL_2")
range(z_PESL_2)
dim(z_PESL_2)

z_PESL_s = outer(a, b, FUN = "PESL_s")
range(z_PESL_s)
dim(z_PESL_s)

range(z_PESL_2 - z_PESL_s)

z_PESL_2_s = outer(a, b, FUN = "PESL_2_s")
range(z_PESL_2_s)

open3d()
persp3d(a, b, z_PESL_2, col = "red", xlab = "a", ylab = "b", zlab = "PESLs")
persp3d(a, b, z_PESL_s, col = "blue", add = TRUE)
rgl.postscript("figure/noninformative_scale_PESLs.pdf", "pdf")
```

```
open3d()
persp3d(a, b, z_PESL_2_s, col = "green", xlab = "a", ylab = "b", zlab = "")
rgl.postscript("figure/noninformative_scale_PESL_2_s.pdf", "pdf")
```

A.3. MAIN_REAL_DATA.R

```
###################################################
### code chunk number : set your work directory
###################################################
##
## Firstly, set your work directory to contain "main_real_data.R".
## Secondly, make sure there is a folder named "figure"
## in the same directory of "main_real_data.R".
##


####################################################
### code chunk number : monthly, simple return
####################################################
##
##
rm(list=ls(all=TRUE))
source("subfunctions.R")

##
## Install the package for the first time.
## note: omit the 's' in 'https' if you cannot handle https downloads
## install.packages(pkgs = "quantmod",
##                  repos = "https://cloud.r-project.org/", dependencies=TRUE)
library(quantmod)

##
## getSymbols from SP500
##
SP500 = getSymbols("^GSPC", auto.assign=FALSE, from = "2016-07-30",
                   to = "2021-07-30")
head(SP500)

##
## obtain SP500_close and SP500_close_m
##
SP500_close = SP500[, 4]
SP500_close = na.omit(SP500_close) ## omit the NA values
N = length(SP500_close); N
```

```
N_m = floor(N / 20); N_m
SP500_close_m = SP500[20*(1:N_m), 4]

L_SP500_close_m = length(SP500_close_m); L_SP500_close_m
Num = 1:L_SP500_close_m
cbind(Num, SP500_close_m)

##
## calculte simple return SP500_return_m
##
SP500_return_m = SP500_close_m[2:N_m]
SP500_return_m[, 1] =
(as.vector(SP500_close_m[2:N_m]) - as.vector(SP500_close_m[1:(N_m-1)]))
 / as.vector(SP500_close_m[1:(N_m-1)])
dimnames(SP500_return_m)[[2]] = "Return"
SP500_return_m

##
## p-value = 0.643 between 2020-04-24 and 2021-07-30
## SP500_return_m[46:L]
##
L = length(SP500_return_m); L
p_value_return_m = numeric(L)
names(p_value_return_m) = 1:L
for (i in 1:L){
     p_value_return_m[i] = shapiro.test(as.vector(SP500_return_m[i:L]))$p.value
}
round(p_value_return_m, 3)

cbind(1:L, SP500_return_m)

SP500_return_m[46:L]

res_return_m = shapiro.test(as.vector(SP500_return_m[46:L])); res_return_m

##
## plot SP500_close_m
##
dev.new()
SP500_close_prices = SP500_close_m[(46 + 1):L_SP500_close_m]
plot(as.vector(SP500_close_prices), type = "l",
     ylab = "SP500 monthly close prices", lwd = 4)
savePlot(filename = "figure/SP500_monthly_close_prices", type = c("pdf"))
savePlot(filename = "figure/SP500_monthly_close_prices", type = c("jpg"))
```

```
##
## plot SP500_return_m[46:L]
##
dev.new()
plot(as.vector(SP500_return_m[46:L]), type = "l",
     ylab = "SP500 monthly simple returns", lwd = 4)
savePlot(filename = "figure/SP500_monthly_simple_returns", type = c("pdf"))
savePlot(filename = "figure/SP500_monthly_simple_returns", type = c("jpg"))

##
## Histogram of SP500 monthly simple returns
##
dev.new()
Return_m = as.vector(SP500_return_m[46:L])
col = c("blue", "red")
lty = 1:2
hist(Return_m, probability = TRUE,
    main = paste("Histogram of" , "SP500 monthly simple returns"),
    xlab = "SP500 monthly simple returns", ylim = c(0, 25))
lines(density(Return_m), lty = lty[1], col = col[1], lwd = 4)
x = seq(-0.3, 0.3, by = 0.001)
y = dnorm(x, mean(Return_m), sd(Return_m))
lines(x, y,              lty = lty[2], col = col[2], lwd = 4)
leg.txt = c( "Density estimation","Normal approximation")
legend("topright", legend = leg.txt, lty = lty, col = col, lwd = 2)
savePlot(filename = "figure/Histogram_SP500_monthly_simple_returns",
         type = c("pdf"))
savePlot(filename = "figure/Histogram_SP500_monthly_simple_returns",
         type = c("jpg"))

##
## compute the estimators and the PESLs
##
## x is the data. In some occasions, SP500 = getSymbols(...) does not work, then
## we could use "x.csv" directly.
##
x = as.vector(SP500_return_m[46:L]); x
write.csv(x, file="x.csv")
mu = mean(x); mu
n = length(x); n
alpha = 1
beta = 1

res_conj_var = Compute_PostDist_Estimators_PESLs_conjugate_variance(
               mu, alpha, beta, n, x); res_conj_var[2:5]
```

```
res_conj_sca = Compute_PostDist_Estimators_PESLs_conjugate_scale(
               mu, alpha, beta, n, x); res_conj_sca[2:5]
res_noni_var = Compute_PostDist_Estimators_PESLs_noninformative_variance(
               mu, n, x); res_noni_var[2:5]
res_noni_sca = Compute_PostDist_Estimators_PESLs_noninformative_scale(
               mu, n, x); res_noni_sca[2:5]

##
## Generate_Matrix_Latex
##
A = matrix(0, nrow = 4, ncol = 4)
A[, 1] = c(res_conj_var$delta_s, res_conj_var$delta_2,
           res_conj_var$PESL_s, res_conj_var$PESL_2)
A[, 2] = c(res_conj_sca$delta_s, res_conj_sca$delta_2,
           res_conj_sca$PESL_s, res_conj_sca$PESL_2)
A[, 3] = c(res_noni_var$delta_s, res_noni_var$delta_2,
           res_noni_var$PESL_s, res_noni_var$PESL_2)
A[, 4] = c(res_noni_sca$delta_s, res_noni_sca$delta_2,
           res_noni_sca$PESL_s, res_noni_sca$PESL_2)
A = round(A, 6); A
A = format(A, nsmall = 6); A
Generate_Matrix_Latex(A)
```

A.4. SUBFUNCTIONS.R

```
Compute_PostDist_Estimators_PESLs_conjugate_variance = function(
mu, alpha, beta, n, x){

alpha1 = alpha + n/2
beta1 = 1 / (1/beta + sum((x - mu)^2) / 2)
PostDist_theta = 1 / rgamma(n = 1000, shape = alpha1, scale = beta1)

E1 = 1 / ((alpha1 - 1) * beta1) ## alpha1 > 1
E2 = alpha1 * beta1
E3 = - log(beta1) - digamma(alpha1)

delta_s = 1 / E2
delta_2 = E1
PESL_s = log(E2) + E3
PESL_2 = E1 * E2 - log(E1) + E3 - 1

res = list(
PostDist_theta = PostDist_theta,
delta_s = delta_s,
delta_2 = delta_2,
```

```
PESL_s = PESL_s,
PESL_2 = PESL_2
)
}

Compute_PostDist_Estimators_PESLs_conjugate_scale = function(
mu, alpha, beta, n, x){

alpha1 = alpha + n/2
beta1 = 1 / (1/beta + sum((x - mu)^2) / 2)
PostDist_theta = sqrt(1 / rgamma(n = 1000, shape = alpha1, scale = beta1))

E1 = gamma(alpha1 - 1/2) / (gamma(alpha1) * sqrt(beta1)) ## alpha1 > 1/2
E2 = gamma(alpha1 + 1/2) * sqrt(beta1) / gamma(alpha1)
E3 = - log(beta1)/2 - digamma(alpha1)/2

delta_s = 1 / E2
delta_2 = E1
PESL_s = log(E2) + E3
PESL_2 = E1 * E2 - log(E1) + E3 - 1

res = list(
PostDist_theta = PostDist_theta,
delta_s = delta_s,
delta_2 = delta_2,
PESL_s = PESL_s,
PESL_2 = PESL_2
)
}

Compute_PostDist_Estimators_PESLs_noninformative_variance = function(mu, n, x){

alpha1 = n/2
beta1 = 2 / sum((x - mu)^2)
PostDist_theta = 1 / rgamma(n = 1000, shape = alpha1, scale = beta1)

E1 = 1 / ((alpha1 - 1) * beta1) ## alpha1 > 1
E2 = alpha1 * beta1
E3 = - log(beta1) - digamma(alpha1)

delta_s = 1 / E2
delta_2 = E1
PESL_s = log(E2) + E3
PESL_2 = E1 * E2 - log(E1) + E3 - 1
```

```
res = list(
PostDist_theta = PostDist_theta,
delta_s = delta_s,
delta_2 = delta_2,
PESL_s = PESL_s,
PESL_2 = PESL_2
)
}

Compute_PostDist_Estimators_PESLs_noninformative_scale = function(mu, n, x){

alpha1 = n/2
beta1 = 2 / sum((x - mu)^2)
PostDist_theta = sqrt(1 / rgamma(n = 1000, shape = alpha1, scale = beta1))

E1 = gamma(alpha1 - 1/2) / (gamma(alpha1) * sqrt(beta1)) ## alpha1 > 1/2
E2 = gamma(alpha1 + 1/2) * sqrt(beta1) / gamma(alpha1)
E3 = - log(beta1)/2 - digamma(alpha1)/2

delta_s = 1 / E2
delta_2 = E1
PESL_s = log(E2) + E3
PESL_2 = E1 * E2 - log(E1) + E3 - 1

res = list(
PostDist_theta = PostDist_theta,
delta_s = delta_s,
delta_2 = delta_2,
PESL_s = PESL_s,
PESL_2 = PESL_2
)
}

plot_estimator = function(delta_s, delta_2, parameter, para_name,
legend_pos = "topleft"){

dev.new()
col = c("blue", "red")
lty = 1:2
xlim = range(parameter)
ylim = range(delta_s, delta_2)
plot(parameter, delta_s, type = "n", xlim = xlim, ylim = ylim,
     xlab = substitute(parameter), ylab = "Estimator")
lines(parameter, delta_s, lty = lty[1], col = col[1], lwd = 4)
lines(parameter, delta_2, lty = lty[2], col = col[2], lwd = 4)
```

```
leg.txt = c("delta_s", "delta_2")
legend(x = legend_pos, legend = leg.txt, lty = lty, col = col, lwd = 2)
savePlot(filename = paste("figure/", para_name, "_change_delta", sep = ""),
         type = c("pdf"))
savePlot(filename = paste("figure/", para_name, "_change_delta", sep = ""),
         type = c("jpg"))
}

plot_PESL = function(PESL_s, PESL_2, parameter, para_name,
legend_pos = "topleft"){

dev.new()
col = c("blue", "red")
lty = 1:2
xlim = range(parameter)
ylim = range(PESL_s, PESL_2)
plot(parameter, PESL_s, type = "n", xlim = xlim, ylim = ylim,
     xlab = substitute(parameter), ylab = "PESL")
lines(parameter, PESL_s, lty = lty[1], col = col[1], lwd = 4)
lines(parameter, PESL_2, lty = lty[2], col = col[2], lwd = 4)
leg.txt = c("PESL_s", "PESL_2")
legend(x = legend_pos, legend = leg.txt, lty = lty, col = col, lwd = 2)
savePlot(filename = paste("figure/", para_name, "_change_PESL", sep = ""),
         type = c("pdf"))
savePlot(filename = paste("figure/", para_name, "_change_PESL", sep = ""),
         type = c("jpg"))
}

Generate_Matrix_Latex = function(A){

m = nrow(A)
n = ncol(A)

C = rep("c", n, sep=""); C
temp = "[c]{"
for (i in 1:n){
temp = paste(temp, C[i], sep="")
}
temp = paste(temp, "}", sep="")

B = matrix("0", nrow = m, ncol = 2*n); B
for (i in 1:m){
    for (j in 1:n){
        B[i, 2*j-1] = A[i, j]
        B[i, 2*j]   = "&"
```

```
    }
}
B[, 2*n] = "\\\\"
B[m, 2*n] = ""
B

One = "\\begin{tabular}"
Two = temp
Three = B
Four = "\\end{tabular}"

cat(One, "\n", Two, "\n"); for (i in 1:m) cat(B[i, ], "\n");  cat(Four, "\n")
}

detail = function(x){

res = list(x=x,
       isS4=isS4(x),
       is.object=is.object(x),
       class=class(x),
       attributes=attributes(x))
res
}
```
