# Supplementary material for: The Bayes Estimators of the Variance and Scale Parameters of the Normal Model With a Known Mean for the Conjugate and Noninformative Priors Under Stein’s Loss
Source: Front Big Data. 2022 Jan 3;4:763925. doi: 10.3389/fdata.2021.763925 (PMC8763389; doi:10.3389/fdata.2021.763925)
Supplement: Supplementary file 2 [file DataSheet1.pdf]

frontiers

Supplementary Material

This is the supplemental file of the paper. The supplement is written for the authors, the reviewers, and the readers to check the correctness of the derivations and to replicate the results.

1 THE CALCULATIONS OF THE QUANTITIES AND EXPRESSIONS OF THE SCALE PARAMETER FOR THE CONJUGATE PRIOR

Since $X|\sigma \sim N\left(\mu, \sigma^2\right)$, we have

$$f\left(x|\sigma\right) = \frac{1}{\sqrt{2\pi}\sigma} \exp\left(-\frac{\left(x-\mu\right)^2}{2\sigma^2}\right)$$

and thus

$$\begin{aligned} f\left(\boldsymbol{x}|\sigma\right) &= \prod_{i=1}^{n} f\left(x_i|\sigma\right) = \prod_{i=1}^{n} \frac{1}{\sqrt{2\pi}\sigma} \exp\left(-\frac{\left(x_i-\mu\right)^2}{2\sigma^2}\right) \\ &\propto \frac{1}{\sigma^n} \exp\left(-\frac{1}{2\sigma^2}\sum_{i=1}^{n}\left(x_i-\mu\right)^2\right) \\ &\sim SRIG\left(*,*\right). \end{aligned}$$

Therefore, we guess that the conjugate prior of σ is $\pi_c\left(\sigma\right) \sim SRIG\left(\alpha,\beta\right)$, that is,

$$\pi_c\left(\sigma\right) = \frac{2}{\Gamma\left(\alpha\right)\beta^\alpha}\left(\frac{1}{\sigma}\right)^{2\alpha+1} \exp\left(-\frac{1}{\beta\sigma^2}\right).$$

Consequently,

$$\begin{aligned} \pi_c\left(\sigma|\boldsymbol{x}\right) &\propto f\left(\boldsymbol{x}|\sigma\right)\pi_c\left(\sigma\right) \\ &\propto \frac{1}{\sigma^n} \exp\left(-\frac{1}{2\sigma^2}\sum_{i=1}^{n}\left(x_i-\mu\right)^2\right)\left(\frac{1}{\sigma}\right)^{2\alpha+1} \exp\left(-\frac{1}{\beta\sigma^2}\right) \\ &= \left(\frac{1}{\sigma}\right)^{2\alpha+n+1} \exp\left(-\frac{1}{\sigma^2}\left[\frac{1}{2}\sum_{i=1}^{n}\left(x_i-\mu\right)^2+\frac{1}{\beta}\right]\right) \\ &= \left(\frac{1}{\sigma}\right)^{2\alpha^*+1} \exp\left(-\frac{1}{\beta^*\sigma^2}\right) \\ &\sim SRIG\left(\alpha^*,\beta^*\right), \end{aligned}$$

where

$$\alpha^* = \alpha + \frac{n}{2} \text{ and } \beta^* = \left[\frac{1}{2}\sum_{i=1}^{n}\left(x_i-\mu\right)^2+\frac{1}{\beta}\right]^{-1}.$$

The rest quantities and expressions of the scale parameter $\theta=\sigma$ for the conjugate prior follow those of the scale parameter for the noninformative priors, as given in Table 2 of the manuscript, with $\tilde{\alpha}$ and $\tilde{\beta}$ replaced by α^* and β^*, since their forms of posterior distributions are the same. These quantities and expressions of the scale parameter $\theta=\sigma$ for the conjugate prior are given below:

$$\begin{aligned}
\mathrm{E}^{\pi_c}\left(\sigma|\boldsymbol{x}\right) &= \frac{\Gamma\left(\alpha^*-\frac{1}{2}\right)}{\Gamma\left(\alpha^*\right)\beta^{*\frac{1}{2}}}, \text{ for } \alpha^*>\frac{1}{2}, \\
\mathrm{E}^{\pi_c}\left(\frac{1}{\sigma}|\boldsymbol{x}\right) &= \frac{\Gamma\left(\alpha^*+\frac{1}{2}\right)\beta^{*\frac{1}{2}}}{\Gamma\left(\alpha^*\right)}, \\
\mathrm{E}^{\pi_c}\left(\log\sigma|\boldsymbol{x}\right) &= -\frac{1}{2}\log\beta^*-\frac{1}{2}\psi\left(\alpha^*\right),
\end{aligned}$$

$$\begin{aligned}
\delta_s^{\pi_c,\sigma}\left(\boldsymbol{x}\right) &= \frac{\Gamma\left(\alpha^*\right)}{\Gamma\left(\alpha^*+\frac{1}{2}\right)\beta^{*\frac{1}{2}}}, \\
\delta_2^{\pi_c,\sigma}\left(\boldsymbol{x}\right) &= \frac{\Gamma\left(\alpha^*-\frac{1}{2}\right)}{\Gamma\left(\alpha^*\right)\beta^{*\frac{1}{2}}}, \text{ for } \alpha^*>\frac{1}{2}, \\
PESL_s^{\pi_c,\sigma}\left(\boldsymbol{x}\right) &= \log\Gamma\left(\alpha^*+\frac{1}{2}\right)-\log\Gamma\left(\alpha^*\right)-\frac{1}{2}\psi\left(\alpha^*\right), \\
PESL_2^{\pi_c,\sigma}\left(\boldsymbol{x}\right) &= \left[\begin{array}{c}\frac{\Gamma\left(\alpha^*-\frac{1}{2}\right)\Gamma\left(\alpha^*+\frac{1}{2}\right)}{\Gamma^2\left(\alpha^*\right)}-1-\log\Gamma\left(\alpha^*-\frac{1}{2}\right) \\ +\log\Gamma\left(\alpha^*\right)-\frac{1}{2}\psi\left(\alpha^*\right)\end{array}\right], \text{ for } \alpha^*>\frac{1}{2}.
\end{aligned}$$

The calculations are complete. □

2 THE CALCULATIONS NEEDED IN PROPOSITION 1

We first calculate $\mathrm{E}\left(\sigma\right)$. We have

$$\begin{aligned}
\mathrm{E}\left(\sigma\right) &= \int_0^\infty \sigma f_\sigma\left(\sigma|\alpha,\beta\right)d\sigma \\
&= \int_0^\infty \sigma\frac{2}{\Gamma\left(\alpha\right)\beta^\alpha}\left(\frac{1}{\sigma}\right)^{2\alpha+1}\exp\left(-\frac{1}{\beta\sigma^2}\right)d\sigma \\
&= \frac{2}{\Gamma\left(\alpha\right)\beta^\alpha}\int_0^\infty\left(\frac{1}{\sigma}\right)^{2\alpha}\exp\left(-\frac{1}{\beta\sigma^2}\right)d\sigma.
\end{aligned}$$

Let $\theta=\sigma^2$. Then $\sigma=\sqrt{\theta}$ and $d\sigma=d\sqrt{\theta}=\frac{1}{2\sqrt{\theta}}d\theta$. Therefore,

$$\begin{aligned}
\mathrm{E}\left(\sigma\right) &= \frac{2}{\Gamma\left(\alpha\right)\beta^\alpha}\int_0^\infty\left(\frac{1}{\theta}\right)^{\alpha}\exp\left(-\frac{1}{\beta\theta}\right)\frac{1}{2\sqrt{\theta}}d\theta \\
&= \frac{1}{\Gamma\left(\alpha\right)\beta^\alpha}\int_0^\infty\left(\frac{1}{\theta}\right)^{\alpha+\frac{1}{2}}\exp\left(-\frac{1}{\beta\theta}\right)d\theta.
\end{aligned}$$

Since

$$\int_0^\infty \frac{1}{\Gamma(\alpha)\beta^\alpha}\left(\frac{1}{\theta}\right)^{\alpha+1}\exp\left(-\frac{1}{\beta\theta}\right)d\theta = 1$$

where the integrand is the pdf of the $IG(\alpha,\beta)$ distribution, we have

$$\int_0^\infty \left(\frac{1}{\theta}\right)^{\alpha+1}\exp\left(-\frac{1}{\beta\theta}\right)d\theta = \Gamma(\alpha)\beta^\alpha,$$

for $\alpha > 0$ and $\beta > 0$. Consequently,

$$\begin{aligned}
\mathrm{E}(\sigma) &= \frac{1}{\Gamma(\alpha)\beta^\alpha}\int_0^\infty \left(\frac{1}{\theta}\right)^{\left(\alpha-\frac{1}{2}\right)+1}\exp\left(-\frac{1}{\beta\theta}\right)d\theta \\
&= \frac{1}{\Gamma(\alpha)\beta^\alpha}\Gamma\left(\alpha-\frac{1}{2}\right)\beta^{\alpha-\frac{1}{2}} \\
&= \frac{\Gamma\left(\alpha-\frac{1}{2}\right)}{\Gamma(\alpha)\beta^{\frac{1}{2}}}, \text{ for } \alpha > \frac{1}{2} \text{ and } \beta > 0.
\end{aligned}$$

Now we calculate $\mathrm{E}\left(\frac{1}{\sigma}\right)$. We have

$$\begin{aligned}
\mathrm{E}\left(\frac{1}{\sigma}\right) &= \int_0^\infty \frac{1}{\sigma} f_\sigma(\sigma|\alpha,\beta)\,d\sigma \\
&= \int_0^\infty \frac{1}{\sigma}\frac{2}{\Gamma(\alpha)\beta^\alpha}\left(\frac{1}{\sigma}\right)^{2\alpha+1}\exp\left(-\frac{1}{\beta\sigma^2}\right)d\sigma \\
&= \frac{2}{\Gamma(\alpha)\beta^\alpha}\int_0^\infty \left(\frac{1}{\sigma}\right)^{2(\alpha+1)}\exp\left(-\frac{1}{\beta\sigma^2}\right)d\sigma.
\end{aligned}$$

Let $\theta = \sigma^2$. Then

$$\begin{aligned}
\mathrm{E}\left(\frac{1}{\sigma}\right) &= \frac{2}{\Gamma(\alpha)\beta^\alpha}\int_0^\infty \left(\frac{1}{\theta}\right)^{\alpha+1}\exp\left(-\frac{1}{\beta\theta}\right)\frac{1}{2\sqrt{\theta}}d\theta \\
&= \frac{1}{\Gamma(\alpha)\beta^\alpha}\int_0^\infty \left(\frac{1}{\theta}\right)^{\left(\alpha+\frac{1}{2}\right)+1}\exp\left(-\frac{1}{\beta\theta}\right)d\theta \\
&= \frac{1}{\Gamma(\alpha)\beta^\alpha}\Gamma\left(\alpha+\frac{1}{2}\right)\beta^{\alpha+\frac{1}{2}} \\
&= \frac{\Gamma\left(\alpha+\frac{1}{2}\right)\beta^{\frac{1}{2}}}{\Gamma(\alpha)}, \text{ for } \alpha > 0 \text{ and } \beta > 0.
\end{aligned}$$

Finally, let us calculate $\mathrm{E}(\log\sigma)$. We have

$$\begin{aligned}\mathrm{E}(\log\sigma) &= \int_0^\infty \log\sigma\cdot f_\sigma(\sigma|\alpha,\beta)\,d\sigma\\ &= \int_0^\infty \log\sigma\cdot\frac{2}{\Gamma(\alpha)\beta^\alpha}\left(\frac{1}{\sigma}\right)^{2\alpha+1}\exp\left(-\frac{1}{\beta\sigma^2}\right)d\sigma\\ &= \frac{2}{\Gamma(\alpha)\beta^\alpha}\int_0^\infty \log\sigma\cdot\left(\frac{1}{\sigma}\right)^{2\alpha+1}\exp\left(-\frac{1}{\beta\sigma^2}\right)d\sigma.\end{aligned}$$

Let $y=\frac{1}{\beta\sigma^2}$. Then $\sigma=\frac{1}{\sqrt{y\beta}}$ and $d\sigma=\frac{1}{\sqrt{\beta}}\left(-\frac{1}{2}\right)y^{-\frac{3}{2}}dy$. Therefore,

$$\begin{aligned}\mathrm{E}(\log\sigma) &= \frac{2}{\Gamma(\alpha)\beta^\alpha}\int_\infty^0 \log(y\beta)^{-\frac{1}{2}}\cdot(y\beta)^{\frac{2\alpha+1}{2}}\exp(-y)\frac{1}{\sqrt{\beta}}\left(-\frac{1}{2}\right)y^{-\frac{3}{2}}dy\\ &= \frac{1}{\Gamma(\alpha)\beta^\alpha}\int_0^\infty\left(-\frac{1}{2}\right)(\log y+\log\beta)\,y^{\alpha+\frac{1}{2}}\beta^{\alpha+\frac{1}{2}}\exp(-y)\,\beta^{-\frac{1}{2}}y^{-\frac{3}{2}}dy\\ &= \left(-\frac{1}{2}\right)\frac{1}{\Gamma(\alpha)}\int_0^\infty(\log y+\log\beta)\,y^{\alpha-1}\exp(-y)\,dy\\ &= \left(-\frac{1}{2}\right)\frac{1}{\Gamma(\alpha)}\left[(\log\beta)\,\Gamma(\alpha)+\int_0^\infty(\log y)\,y^{\alpha-1}\exp(-y)\,dy\right]\\ &= -\frac{1}{2}\log\beta-\frac{1}{2\Gamma(\alpha)}\int_0^\infty\frac{d}{d\alpha}\left(y^{\alpha-1}\exp(-y)\right)dy\\ &= -\frac{1}{2}\log\beta-\frac{1}{2\Gamma(\alpha)}\frac{d}{d\alpha}\int_0^\infty y^{\alpha-1}\exp(-y)\,dy\\ &= -\frac{1}{2}\log\beta-\frac{\Gamma'(\alpha)}{2\Gamma(\alpha)}\\ &= -\frac{1}{2}\log\beta-\frac{1}{2}\psi(\alpha),\ \text{for}\ \alpha>0\ \text{and}\ \beta>0.\end{aligned}$$

The calculations are complete. □

3 THE PROOF OF PROPOSITION 2

The “only if” part is correct by Definition 1. Now we prove the “if” part. If $\sigma=\sqrt{\theta}\sim SRIG(\alpha,\beta)$, then

$$\begin{aligned}f_\theta(\theta|\alpha,\beta) &= f_\sigma(\sigma|\alpha,\beta)\left|\sigma'(\theta)\right|\\ &= \frac{2}{\Gamma(\alpha)\beta^\alpha}\left(\frac{1}{\sqrt{\theta}}\right)^{2\alpha+1}\exp\left(-\frac{1}{\beta\theta}\right)\frac{1}{2\sqrt{\theta}}\\ &= \frac{1}{\Gamma(\alpha)\beta^\alpha}\left(\frac{1}{\theta}\right)^{\alpha+1}\exp\left(-\frac{1}{\beta\theta}\right)\\ &\sim IG(\alpha,\beta).\end{aligned}$$

The proposition is proved. □

4 THE PROOF OF THEOREM 1

First, let us calculate the posterior distribution of $\theta = \sigma^2$, $\pi\left(\theta|\boldsymbol{x}\right)$. Since $X|\theta \sim N\left(\mu, \theta\right)$, we have

$$f\left(x|\theta\right) = \frac{1}{\sqrt{2\pi}\sqrt{\theta}} \exp\left(-\frac{\left(x-\mu\right)^2}{2\theta}\right)$$

and thus

$$f\left(\boldsymbol{x}|\theta\right) = \prod_{i=1}^{n} f\left(x_i|\theta\right) = \prod_{i=1}^{n} \frac{1}{\sqrt{2\pi}\sqrt{\theta}} \exp\left(-\frac{\left(x_i-\mu\right)^2}{2\theta}\right) \propto \theta^{-\frac{n}{2}} \exp\left(-\frac{1}{2\theta}\sum_{i=1}^{n}\left(x_i-\mu\right)^2\right).$$

Therefore,

$$\begin{aligned}\pi\left(\theta|\boldsymbol{x}\right) &\propto f\left(\boldsymbol{x}|\theta\right)\pi\left(\theta\right)\\ &\propto \theta^{-\frac{n}{2}} \exp\left(-\frac{1}{2\theta}\sum_{i=1}^{n}\left(x_i-\mu\right)^2\right)\theta^{-1}\\ &= \left(\frac{1}{\theta}\right)^{\frac{n}{2}+1} \exp\left(-\frac{1}{2\theta}\sum_{i=1}^{n}\left(x_i-\mu\right)^2\right)\\ &\sim IG\left(\tilde{\alpha}, \tilde{\beta}\right),\end{aligned}$$

where

$$\tilde{\alpha} = \frac{n}{2} \text{ and } \tilde{\beta} = \frac{2}{\sum_{i=1}^{n}\left(x_i-\mu\right)^2}.$$

Now we find the posterior distribution of $\theta = \sigma$, $\pi\left(\sigma|\boldsymbol{x}\right)$. Since $X|\sigma \sim N\left(\mu, \sigma^2\right)$, we have

$$f\left(x|\sigma\right) = \frac{1}{\sqrt{2\pi}\sigma} \exp\left(-\frac{\left(x-\mu\right)^2}{2\sigma^2}\right)$$

and thus

$$f\left(\boldsymbol{x}|\sigma\right) = \prod_{i=1}^{n} f\left(x_i|\sigma\right) = \prod_{i=1}^{n} \frac{1}{\sqrt{2\pi}\sigma} \exp\left(-\frac{\left(x_i-\mu\right)^2}{2\sigma^2}\right) \propto \frac{1}{\sigma^n} \exp\left(-\frac{1}{2\sigma^2}\sum_{i=1}^{n}\left(x_i-\mu\right)^2\right).$$

Therefore,

$$\begin{aligned}\pi\left(\sigma|\boldsymbol{x}\right) &\propto f\left(\boldsymbol{x}|\sigma\right)\pi\left(\sigma\right)\\ &\propto \frac{1}{\sigma^n}\exp\left(-\frac{1}{2\sigma^2}\sum_{i=1}^{n}\left(x_i-\mu\right)^2\right)\frac{1}{\sigma}\\ &=\left(\frac{1}{\sigma}\right)^{n+1}\exp\left(-\frac{1}{2\sigma^2}\sum_{i=1}^{n}\left(x_i-\mu\right)^2\right)\\ &\sim SRIG\left(\tilde{\alpha},\tilde{\beta}\right).\end{aligned}$$

The theorem is proved. □

5 THE PROOF OF THE INEQUALITY FOR THE PESLS OF THE VARIANCE PARAMETER

In this section, we will prove

$$\begin{aligned}PESL_s^{\pi,\theta}\left(\boldsymbol{x}\right) &= \log\tilde{\alpha}-\psi\left(\tilde{\alpha}\right)\\ &\leq \frac{1}{\tilde{\alpha}-1}+\log\left(\tilde{\alpha}-1\right)-\psi\left(\tilde{\alpha}\right)=PESL_2^{\pi,\theta}\left(\boldsymbol{x}\right),\end{aligned} \tag{S1}$$

for $\tilde{\alpha}>1$.

The inequality (S1) is equivalent to

$$\log\tilde{\alpha}\leq\frac{1}{\tilde{\alpha}-1}+\log\left(\tilde{\alpha}-1\right)$$

$$\begin{aligned}&\Longleftrightarrow \log\tilde{\alpha}-\log\left(\tilde{\alpha}-1\right)\leq\frac{1}{\tilde{\alpha}-1}\\ &\Longleftrightarrow \log\frac{\tilde{\alpha}}{\tilde{\alpha}-1}\leq\frac{1}{\tilde{\alpha}-1}\\ &\Longleftrightarrow \frac{\tilde{\alpha}}{\tilde{\alpha}-1}\leq\exp\left(\frac{1}{\tilde{\alpha}-1}\right).\end{aligned} \tag{S2}$$

Note that

$$\frac{\tilde{\alpha}}{\tilde{\alpha}-1}=\frac{\tilde{\alpha}-1+1}{\tilde{\alpha}-1}=1+\frac{1}{\tilde{\alpha}-1}.$$

The Taylor series expansion for e^x with $x=\frac{1}{\tilde{\alpha}-1}$ gives

$$\begin{aligned}\exp\left(\frac{1}{\tilde{\alpha}-1}\right)&=1+\frac{1}{\tilde{\alpha}-1}+\frac{1}{2!}\frac{1}{\left(\tilde{\alpha}-1\right)^2}+\cdots\\ &>1+\frac{1}{\tilde{\alpha}-1}=\frac{\tilde{\alpha}}{\tilde{\alpha}-1},\text{ for }\tilde{\alpha}>1.\end{aligned}$$

Therefore, (S2) is correct and (S1) is proved. □

6 THE PROOF OF THE INEQUALITY FOR THE BAYES ESTIMATORS OF THE SCALE PARAMETER

In this section, we will prove

$$\delta_s^{\pi,\sigma}(\boldsymbol{x}) = \frac{\Gamma(\tilde{\alpha})}{\Gamma\left(\tilde{\alpha}+\frac{1}{2}\right)\tilde{\beta}^{\frac{1}{2}}} < \frac{\Gamma\left(\tilde{\alpha}-\frac{1}{2}\right)}{\Gamma(\tilde{\alpha})\tilde{\beta}^{\frac{1}{2}}} = \delta_2^{\pi,\sigma}(\boldsymbol{x}), \tag{S3}$$

for $\tilde{\alpha} > \frac{1}{2}$.

The inequality (S3) is equivalent to

$$\frac{\Gamma(\tilde{\alpha})}{\Gamma\left(\tilde{\alpha}+\frac{1}{2}\right)} < \frac{\Gamma\left(\tilde{\alpha}-\frac{1}{2}\right)}{\Gamma(\tilde{\alpha})}. \tag{S4}$$

Let

$$G(x) = \frac{\Gamma(x)}{\Gamma\left(x+\frac{1}{2}\right)}, \text{ for } x > 0.$$

Then

$$G'(x) = \left(\frac{\Gamma(x)}{\Gamma\left(x+\frac{1}{2}\right)}\right)' = \frac{\Gamma'(x)\Gamma\left(x+\frac{1}{2}\right) - \Gamma(x)\Gamma'\left(x+\frac{1}{2}\right)}{\Gamma^2\left(x+\frac{1}{2}\right)} < 0$$

$$\iff \Gamma'(x)\Gamma\left(x+\frac{1}{2}\right) < \Gamma(x)\Gamma'\left(x+\frac{1}{2}\right)$$

$$\iff \psi(x) = \frac{\Gamma'(x)}{\Gamma(x)} < \frac{\Gamma'\left(x+\frac{1}{2}\right)}{\Gamma\left(x+\frac{1}{2}\right)} = \psi\left(x+\frac{1}{2}\right).$$

We claim that

$$\psi'(x) = \frac{d^2}{dx^2}\log\Gamma(x) = \mathrm{trigamma}(x) > 0, \text{ for } x > 0,$$

which can be checked in R software by plotting the trigamma function over the intervals $[0.001, 0.1]$, $[0.1, 10]$, and $[10, 1000]$. Alternatively, we can prove that $\psi'(x) > 0$ by exploiting the formula (6.4.1)

$$\psi^{(n)}(z) = (-1)^{n+1}\int_0^\infty \frac{t^n e^{-zt}}{1-e^{-t}}dt, \ (\mathrm{Re}(z) > 0),$$

in Abramowitz and Stegun (1970). Let $n = 1$ and $z = x > 0$ in the above formula, we obtain

$$\psi'(x) = \int_0^\infty \frac{te^{-xt}}{1-e^{-t}}dt > 0,$$

since the integrand is positive for all $t > 0$. Therefore, $\psi(x)$ is an increasing function, and thus $\psi(x) < \psi\left(x+\frac{1}{2}\right)$ and $G'(x) < 0$. Consequently, $G(x)$ is a decreasing function for $x > 0$. Therefore, (S4) is correct and (S3) is proved. □

7 THE PROOF OF THE INEQUALITY FOR THE PESLS OF THE SCALE PARAMETER

In this section, we will prove

$$\begin{aligned} & PESL_s^{\pi,\sigma}(\boldsymbol{x}) \\ & = \log\Gamma\left(\tilde{\alpha}+\frac{1}{2}\right) - \log\Gamma\left(\tilde{\alpha}\right) - \frac{1}{2}\psi\left(\tilde{\alpha}\right) \\ & \leq \frac{\Gamma\left(\tilde{\alpha}-\frac{1}{2}\right)\Gamma\left(\tilde{\alpha}+\frac{1}{2}\right)}{\Gamma^2\left(\tilde{\alpha}\right)} - 1 - \log\Gamma\left(\tilde{\alpha}-\frac{1}{2}\right) + \log\Gamma\left(\tilde{\alpha}\right) - \frac{1}{2}\psi\left(\tilde{\alpha}\right) \\ & = PESL_2^{\pi,\sigma}(\boldsymbol{x}), \end{aligned} \tag{S5}$$

for $\tilde{\alpha} > \frac{1}{2}$.

The inequality (S5) is equivalent to

$$\begin{aligned} \log\Gamma\left(\tilde{\alpha}+\frac{1}{2}\right) + \log\Gamma\left(\tilde{\alpha}-\frac{1}{2}\right) - 2\log\Gamma\left(\tilde{\alpha}\right) \leq \frac{\Gamma\left(\tilde{\alpha}-\frac{1}{2}\right)\Gamma\left(\tilde{\alpha}+\frac{1}{2}\right)}{\Gamma^2\left(\tilde{\alpha}\right)} - 1 \\ \Longleftrightarrow \log\frac{\Gamma\left(\tilde{\alpha}-\frac{1}{2}\right)\Gamma\left(\tilde{\alpha}+\frac{1}{2}\right)}{\Gamma^2\left(\tilde{\alpha}\right)} \leq \frac{\Gamma\left(\tilde{\alpha}-\frac{1}{2}\right)\Gamma\left(\tilde{\alpha}+\frac{1}{2}\right)}{\Gamma^2\left(\tilde{\alpha}\right)} - 1. \end{aligned} \tag{S6}$$

Standard Calculus textbooks have the Taylor series expansion for $\log(1+x)$ with x near 0 which is given by

$$\log(1+x) = \sum_{n=1}^{\infty}(-1)^{n-1}\frac{x^n}{n} = x - \frac{x^2}{2} + \frac{x^3}{3} - \cdots.$$

Let $u = 1 + x \approx 1$. Then we obtain a Taylor series expansion for $\log u$ with u near 1 which is given by

$$\begin{aligned} \log u &= \sum_{n=1}^{\infty}(-1)^{n-1}\frac{(u-1)^n}{n} = (u-1) - \frac{(u-1)^2}{2} + \frac{(u-1)^3}{3} - \cdots \\ &\leq u - 1, \end{aligned} \tag{S7}$$

since $u - 1 \approx 0$ and the alternating terms are decreasing in magnitude. Let

$$u = \frac{\Gamma\left(\tilde{\alpha}-\frac{1}{2}\right)\Gamma\left(\tilde{\alpha}+\frac{1}{2}\right)}{\Gamma^2\left(\tilde{\alpha}\right)} \approx 1.$$

By (S7), (S6) is correct and (S5) is proved. □

8 MORE RESULTS FOR THE REAL DATA EXAMPLE

In this section, for the real data example, we will present more results which are not presented in the main text due to space limitations.

The data of the S&P 500 Monthly Simple Returns (MSRs) during 2020-04-24 and 2021-07-02 (henceforth, the period) are summarized in Table S1.

Table S1. The data of the S&P 500 MSRs during the period.

Date	MSRs
2020-04-24	0.078579626
2020-05-22	0.041847318
2020-06-22	0.054952768
2020-07-21	0.044722963
2020-08-18	0.040671715
2020-09-16	−0.001265580
2020-10-14	0.030477104
2020-11-11	0.024075075
2020-12-10	0.026714042
2021-01-11	0.035852350
2021-02-09	0.029376665
2021-03-10	−0.003175451
2021-04-08	0.050877027
2021-05-06	0.025493254
2021-06-04	0.006728362
2021-07-02	0.028948673

The p-value of the S&P 500 monthly simple returns during the period by the Shapiro-Wilk normality test is $0.6431 > 0.05$, which means that the data can be regarded as normal.

The S&P 500 monthly close prices during the period are depicted in Figure S1. From the figure, we see that the S&P 500 monthly close prices increase a lot during the period.

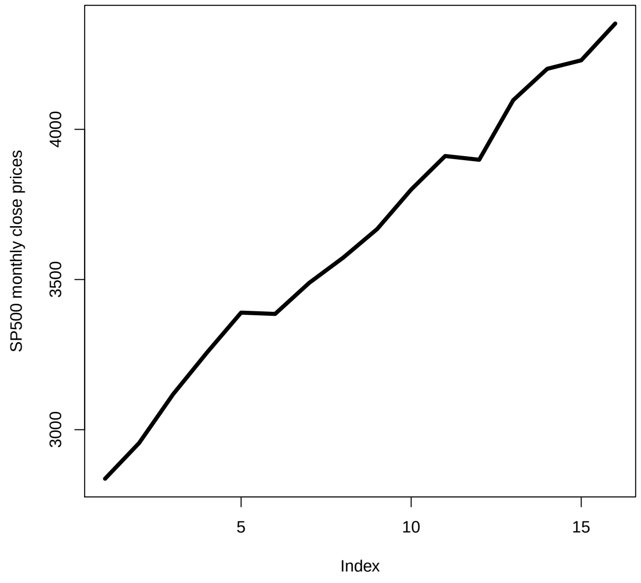

Figure S1. The S&P 500 monthly close prices during the period.

The S&P 500 monthly simple returns during the period are depicted in Figure S2. From the figure, we see that the S&P 500 monthly simple returns fluctuate, and the returns are positive except two occasions.

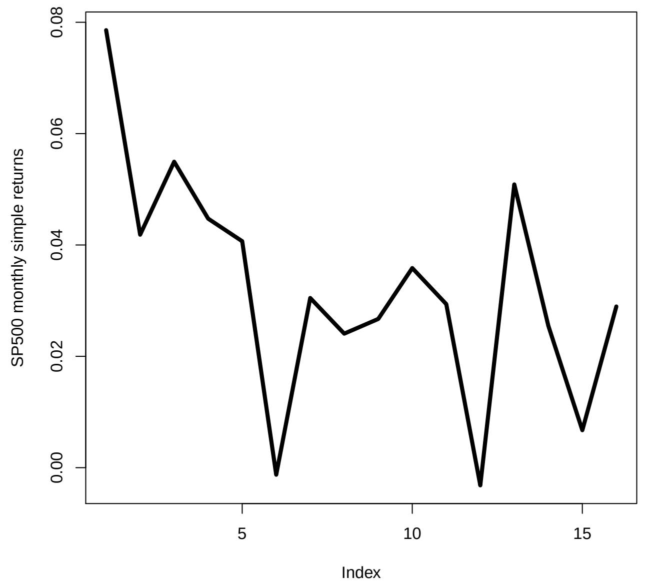

Figure S2. The S&P 500 monthly simple returns during the period.

The histogram of the S&P 500 monthly simple returns during the period are depicted in Figure S3. From the figure, we see that the density estimation curve of the S&P 500 monthly simple returns are roughly bell shaped, and density can be reasonably approximated by a normal distribution.

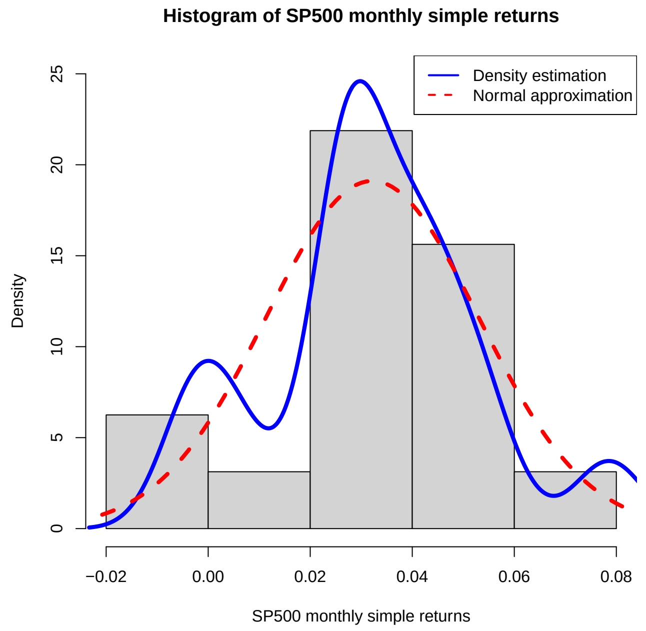

Figure S3. The histogram of the S&P 500 monthly simple returns during the period.

REFERENCES

Abramowitz M, Stegun IA. *Handbook of Mathematical Functions* (New York: United States Government Printing Office) (1970). Ninth Printing edition.
